# Chemosensory protein 3 is a brain host factor for the induction of enhanced-locomotory activity in the BmNPV-silkworm infection model

**DOI:** 10.1371/journal.ppat.1013701

**Published:** 2025-12-01

**Authors:** Min Feng, Shigang Fei, Athanasios Papakyriakou, Eleanna Christodoulou, Wenxuan Lai, Wenjie Luo, Junming Xia, Yigui Huang, Spyros E. Zographos, Luc Swevers, Jingchen Sun

**Affiliations:** 1 Guangdong Provincial Key Laboratory of Agro-animal Genomics and Molecular Breeding, Guangdong Sericulture Engineering Research Center, College of Animal Science, South China Agricultural University, Guangzhou, China; 2 Institute of Biosciences and Applications, National Centre for Scientific Research “Demokritos”, Athens, Greece; 3 Institute of Chemical Biology, National Hellenic Research Foundation, Athens, Greece; 4 Section of Pharmacognosy and Chemistry of Natural Products, Department of Pharmacy, National and Kapodistrian University of Athens, Athens, Greece; 5 Insect Molecular Genetics and Biotechnology, Institute of Biosciences & Applications, National Centre for Scientific Research “Demokritos”, Athens, Greece; Wuhan Institute of Virology, Chinese Academy of Sciences(CAS), CHINA

## Abstract

Although it is reported that the *protein tyrosine phosphatase* gene of baculovirus (group I nucleopolyhedrovirus) can induce enhanced locomotory activity (ELA) in caterpillars, our understanding of the host factors that are involved in the regulation of the behavioral change is still limited. Previously, single-nucleus RNA sequencing (snRNA-seq) was used to identify 19 distinct clusters representing Kenyon cell, glial cell, olfactory projection neuron, optic lobes neuron, hemocyte, muscle cell types and other unannotated cells in the silkworm larvae brains. Analysis of viral transcriptomes in each brain cell subset revealed that all brain cells could be infected by Bombyx mori nucleopolyhedrovirus (BmNPV) at 96 hours post infection but infection occurred at low levels. Furthermore, we found that *chemosensory protein 3* (*CSP3*), encoding a small secreted protein that is possibly implicated in the transport of semiochemicals, was significantly up-regulated after BmNPV infection in most of the brain cell clusters. Knockdown of *BmCSP3* resulted in significantly reduced ELA in BmNPV-infected silkworm larvae. In parallel, targeted metabolomics revealed significant shifts in the abundance of specific lipids and neurotransmitters. Subsequently, structural modeling and molecular dynamics experiments indicated that CSP3 has a large hydrophobic pocket that manifests significant flexibility and likely can accommodate divergent ligand structures or mixtures of them, including known neurotransmitters of the brain and (lyso)glycerophospholipids from larval head samples. *In vitro* binding assays have confirmed the interaction of several neurotransmitters and an eicosanoid to purified BmCSP3 protein. Our study provides insights into the regulation of insect behavior following analysis of viral infection at the single-cell transcriptome level and reveals an unexpected function for CSP proteins in the insect brain.

## Author summary

Baculoviruses, a diverse group of insect-specific DNA viruses, have long been known to induce hyperactive behavior known as enhanced locomotory activity (ELA) and tree-top disease in their lepidopteran hosts. However, our understanding of the host factors that are involved in the hyperactive behavior induced by baculoviruses is still limited. Here, chemosensory protein 3 (CSP3), belonging to a class of secreted proteins known to bind hydrophobic ligands, was identified through single-nucleus RNA sequencing (snRNA-seq) of BmNPV-infected brain tissue as a candidate host factor for induction of ELA. Knockdown of *BmCSP3* resulted in significantly reduced ELA in BmNPV-infected silkworm larvae. *In silico* modeling indicates that BmCSP3 has a large and flexible ligand binding cavity that can accommodate complex lipid molecules. While after infection of silkworm larvae with BmNPV significant changes occur in the lipids and neurotransmitters of head extracts, *in vitro* binding assays confirmed the interaction of several neurotransmitters and an eicosanoid to purified BmCSP3 protein. Our study identified specific CSPs as molecular targets involved in insect brain function and paves the way for biotechnological applications to manipulate insect behavior.

## Introduction

The insect brain, constituted by the cerebral ganglia and the sub-oesophageal ganglion [1], plays crucial roles in the regulation of development and growth as a main neuro-endocrine organ, while all types of behavior are also under its central control [2,3]. The insect brain is often used as an important model system for a comprehensive investigation of neuronal development and function due to its lower number of nerve cells and reduced complexity. However, understanding the molecular, genetic, and cellular mechanisms that underlie brain organization and function still remains one of the most challenging problems of neurobiology. Until a few years ago, the diversity of cell types and their regulatory states in the brain remained largely unknown. With the development of single-cell RNA sequencing (scRNA-seq) and single-nucleus RNA sequencing (snRNA-seq) technology, the cellular landscape of brain tissue has already been reported in different vertebrate and invertebrate species such as mouse [4], zebrafish [5], grouper fish [6], *Drosophila* [7–9], the mosquito *Aedes aegypti* [10] and the domesticated silkworm (*Bombyx mori*) [11]. The single- nucleus transcriptome data of the brain are expected to describe in great detail the response of different brain cells to external stimuli, which will be beneficial for the elucidation of the regulation of brain function.

For the discovery of the mechanisms of brain function, behavior manipulation by parasites offers a unique approach as has been demonstrated in several parasite-insect models that have comprised viruses, bacteria, fungi, apicomplexans, diverse worms and parasitoid wasps [12]. The silkworm and other caterpillars from agricultural pests have attracted interest for studying the central control of behavior by the brain because of their infection with baculoviruses, a diverse group of insect-specific DNA viruses. Baculoviruses have long been known to induce hyperactive behavior in their lepidopteran hosts, which ultimately can result in their migration to the upper plant foliage during the very late stage of infection (just prior to death), known as “Wipfelkrankheit” or tree-top disease [13,14]. Generally, two types of behavioral changes occur during the late stages of baculovirus infection: (1) horizontal hyperactivity, called enhanced locomotory activity (ELA), typically occurring around 4 days post infection for the BmNPV-silkworm infection model [15]; and (2) vertical movement, manifested as climbing behavior or tree-top disease [16]. In the case of larvae of the domesticated silkworm, *B. mori*, climbing behavior is difficult to observe because of weakness in the abdominal legs [17] and research has focused on horizontal movements during ELA [18].

Overall, the molecular mechanisms that underlie the manipulation of host behavior are not very well known [19,20]. Since the brain is a behavioral regulatory center, baculoviruses may affect the behavior of its host mainly by modifying brain activity. For a direct manipulation of brain activity, it is considered that infection of the brain cells by the virus is required. The research on changes in behavior therefore has focused on the interaction between the virus and the host brain [2,21–23]. In terms of viral factors, the baculovirus gene *protein tyrosine phosphatase* (*ptp*) has been identified as a key player for inducing hyperactive behavior following Autographa californica multiple nucleopolyhedrovirus (AcMNPV) and Bombyx mori nucleopolyhedrovirus (BmNPV) infection [14,15,24]. However, the mechanisms clearly differ between the two baculoviruses since phosphatase activity of PTP is required for AcMNPV but not for BmNPV [14,25], while PTP is a structural component of the virions of both viruses [24,26]. Furthermore, the *ptp* gene is only present in group I NPVs (such as AcMNPV and BmNPV) and ELA behavior in group II NPVs (such as Mamestra brassicae NPV; [16]) therefore must have other genetic requirements. By contrast, climbing behavior (tree-top disease) was not affected by *ptp* in AcMNPV infecting *Spodoptera exigua* and BmNPV infecting *B. mandarina* [17,27], therefore demonstrating the independent regulation of the two behaviors (ELA and climbing behavior). A functional role for the baculovirus gene *ecdysteroid UDP glucosyl transferase* (*egt*) in climbing behavior was demonstrated during infection of larvae of the gypsy moth, *Lymantria dispar*, by LdMNPV [28] but not during infection of *S. exigua* or *Trichoplusia ni* larvae by AcMNPV [29] or infection of *B. mandarina* by BmNPV [17]. Egt activity (resulting in the inactivation of 20-hydroxy-ecdysone (20E)) may regulate climbing behavior indirectly, for instance through interference with the molt or the survival of the larvae [16,30].

While two baculovirus-encoded genes have been implicated in behavioral manipulation, much less information is available about the role of host genes. Different omics approaches have been applied to resolve the mechanism of ELA in BmNPV-infected silkworms [2,21,23] but molecular details underlying BmNPV-induced hyperactive behavior have so far remained elusive. Comparative transcriptomics between brains of non-infected and BmNPV-infected larvae identified hundreds of differentially expressed genes, including candidates for the regulation of locomotory behavior [21]. Another study revealed a negative role for 20E and a positive role for dopamine in the regulation of ELA behavior [22]. The pathways to behavioral manipulation may be complex and involve many mechanisms. With the rapid development of multi-omics approaches, major breakthroughs in the elucidation of pathways of behavioral manipulation by parasites could be realized [31]. In such endeavor, the identification of the cells in the brain that change gene expression following baculovirus infection constitutes particularly important information.

In our previous study, cell subsets of the silkworm brain were identified after snRNA-seq data analysis, which resulted in the definition of 14 distinct cell clusters representing Kenyon cell, glial cell, olfactory projection neuron, optic lobes neuron, hemocyte-like cell, muscle cell types and 5 unidentified cell clusters [11]. Here we report on the application of snRNA-seq data analysis to investigate the potential role of chemosensory proteins (CSPs) in the infected silkworm larval brain with respect to ELA. CSPs, as small secreted proteins, are involved in peripheral olfactory processing and are one of the most important classes of proteins for chemoreception due to their functions as carriers of semiochemicals [32]. The present study, through the identified differential expression of particular *CSP* genes in brain, in combination with gene silencing, behavioral bioassays, metabolomic analysis, *in silico* modeling and *in vitro* binding assays, provides valuable targets for studying the molecular mechanisms involved in ELA behavior that is triggered at the late stages of viral infection.

## Results

### The infection status of silkworm brain cell clusters defined by snRNA-seq

**Fig 1:**
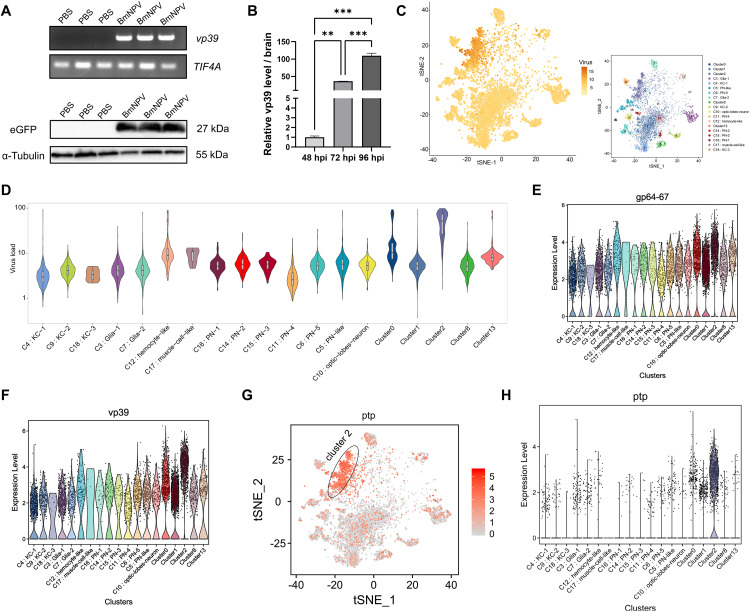
The infection status of each cluster defined by snRNA-seq in BmNPV-infected silkworm larvae brain. **(A)** Agarose gels of RT-PCR products from BmNPV-infected and uninfected brain samples at 96 hpi of BmNPV-eGFP using viral *vp39* gene primers. Western blotting was employed to detect the eGFP reporter protein in silkworm brain at 96 hpi of BmNPV-eGFP. **(B)** The time course of relative expression of virus *vp39* mRNA in the brain at different infection times (72 hpi, 96 hpi), with 48 hpi as the baseline. **(C)** T-distributed stochastic neighbor embedding (t-SNE) visualization of the normalized expression (Z-score) of all viral genes in each BmNPV-infected cell of the brain at 96 hpi. The small image represents the t-SNE plot displaying all identified cell types in BmNPV-infected silkworm larvae brain. **(D)** Violin plot showing the viral load in each brain cell subset across the 0-18 clusters at 96 hpi. **(E-F)** Expression levels of characteristic viral genes including *gp64/67* (envelope gene), *vp39* (encoding the major capsid protein) in brain clusters at 96 hpi. **(G)** t-SNE plot showing distribution of the baculovirus gene *protein tyrosine phosphatase* (*ptp*) in distinct brain cell clusters. **(H)** Violin plot showing the expression levels of *ptp* in brain cell clusters at 96 hpi. Each bar represents the mean ± standard deviation. **p < 0.01, ***P < 0.001.

Firstly, abundant expression of the virus gene (*vp39*) or the eGFP was detected in the brain samples of BmNPV-eGFP-infected silkworm larvae using RT-PCR and Western blot (Fig 1A). This result showed that the silkworm brain was invaded by BmNPV at 96 hpi. Moreover, we found that the expression of *vp39* in the silkworm brain significantly increased at 96 hpi after BmNPV-eGFP infection compared to 48 and 72 hpi (about 109-fold and 3-fold, respectively) (Fig 1B).

Analysis of the snRNA-seq data from our previous study [11] allowed determination of viral gene expression in the different cell subsets. The viral infection status of each cluster in the BmNPV-infected silkworm brain was analyzed following alignment with the BmNPV genome.

At 96 hpi, viral genes were detected in all nuclei indicating that all cells in the brain were infected with BmNPV (Fig 1C). For specific brain cell subsets, the viral load and its variation in the infected cells are presented in the violin plots (Fig 1D). The viral load of infected cells is high only in cluster 2 of the brain at 96 hpi, followed by cluster 12 (hemocyte-like), 17 (muscle cell-like) and the undefined clusters 0 and 13, while the viral load in most cells of the other subsets was low (Fig 1D). The violin plots of Fig 1E and 1F showed that BmNPV genes such as *gp64/67* (encoding envelope fusion protein) and *vp39* (encoding major capsid protein) could be detected in a large number of cells of each cluster in the BmNPV-infected brain at 96 hpi. Interestingly, the expression of *ptp* gene (a key viral gene of BmNPV that induces ELA) was not present in all silkworm brain cells, and in only 22.07% of silkworm brain cells *ptp* expression could be detected (Fig 1G). The violin plot of *ptp* expression also showed that except for a large number of cells in cluster 2, there are very few cells in most other subgroups with abundant expression of the *ptp* gene (Fig 1H).

### Analysis of host factors affecting the occurrence of ELA in the brain of silkworm larvae

**Fig 2:**
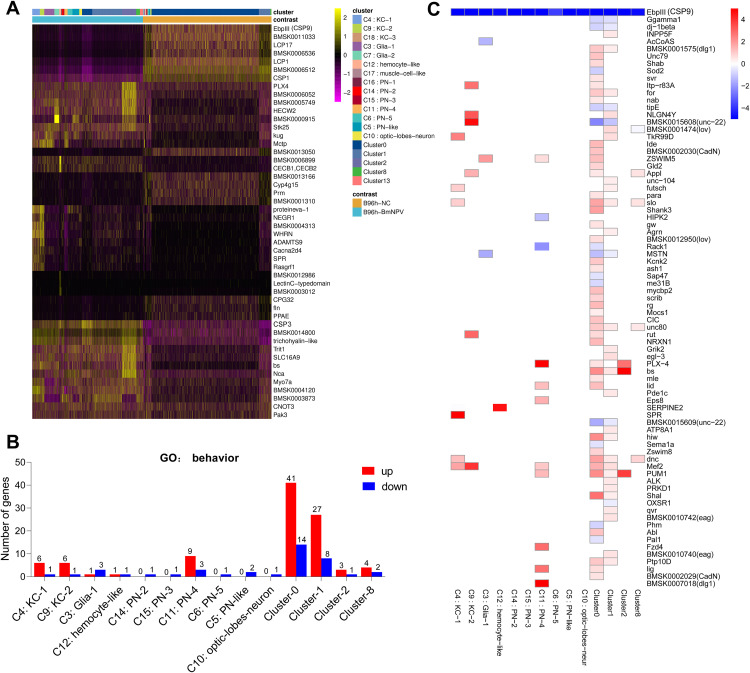
Analysis of host factors affecting the occurrence of silkworm ELA in the brain of silkworm larva. **(A)** Heatmap of top DEGs in BmNPV-infected cells compared to control cells within selected clusters at 96 hpi. The different clusters are color-coded for both BmNPV-infected (left) and NC (right) samples. Genes are indicated by accession numbers in SilkDB 3.0 or by common name abbreviations. **(B)** Histogram showing the number of DEGs enriched for the behavior GO term in each cluster of silkworm brain. **(C)** Heatmap showing that *EbpIII* or *BmCSP9* (BMSK0011182) was the top down-regulated gene after BmNPV infection in all clusters where DEGs are significantly enriched in the GO term “behavior”.

To further screen for host factors that affect the occurrence of silkworm ELA after BmNPV infection, we calculated the differential expression of genes between cell subsets of the BmNPV-infected and non-infected samples using Seurat [33]. Compared to the uninfected brain, it was found that the expression of hundreds of host genes was significantly altered after BmNPV infection in clusters 0, 1 and 11 (PN4), while fewer host genes were affected by viral infection in other subgroups (S1 File). The heatmap of Fig 2A shows the top 50 DEGs in all brain cell subsets at 96 hpi. Notably, the expression of *CSP* genes (*EbpIII* or *BmCSP9*, BMSK0011182), *CSP1* (BMSK0011156) and *CSP3* (BMSK0011153), neuronal genes (*neuropeptide-like precursor 4F* (BMSK0006512), *Nca* (neurocalcin homolog, BMSK0000531), *synapse-associated protein of 47 kDa* (BMSK0004120)) and immune genes (*CECB1,CECB2* (encoding cecropins), BMSK0015392), *lysozyme* (BMSK0006899), *Lectin C-type* (BMSK0015240)) was significantly affected by BmNPV infection in the silkworm brain (Fig 2A).

To further explore the effect of BmNPV infection on silkworm behavior from the perspective of the host, we further analyzed DEGs enriched in the GO term “Behavior” (GO ID (level2):0007610). Except for cluster 0 and cluster 1, the number of DEGs enriched in the term “Behavior” is low in most subgroups of silkworm brain (Fig 2B). Specifically, in cluster 2 with the highest viral load, only three genes including *PUM1* (Pumilio homolog; BMSK0008893), *PLX-4* (plexin homolog; BMSK0010793), and *blistered* (*bs*) (ortholog of serum response factor; BMSK0013093) were significantly up-regulated by BmNPV infection (Fig 2C). Interestingly, the heatmap showed that *BmCSP9* (*EbpIII*, BMSK0011182), related to the GO term “Behavior”, was significantly downregulated after BmNPV infection in all clusters (Fig 2C). In addition, only *BmCSP9* was enriched in the GO term “Behavior” for the *CSP* genes (Fig 2C). This result suggests that CSPs may play an important role in the induction of ELA in silkworms infected with BmNPV at the late stage.

### Changes in expression of *BmCSP9* and *BmCSP1* were unrelated to the occurrence of ELA in silkworm

**Fig 3:**
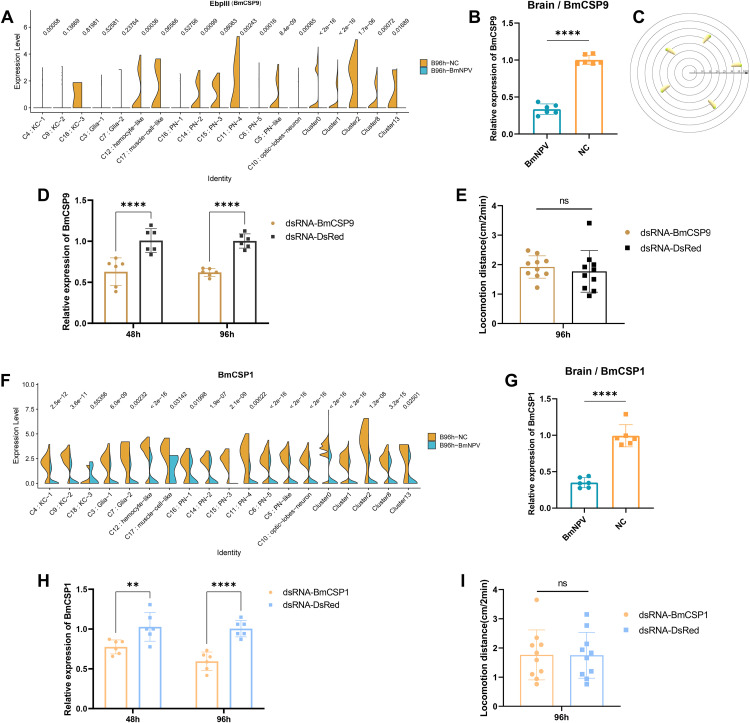
Knockdown of *EbpIII* (*BmCSP9*) and *BmCSP1* in the brain does not affect the regulation of ELA behavior. **(A)** Violin plots of *EbpIII* (*BmCSP9,* BMSK0011182) expression in each brain cluster of BmNPV-infected and uninfected silkworm larvae. **(B)** Quantification of expression of *EbpIII (BmCSP9)* carried out by qPCR using the brain samples collected from BmNPV-infected and uninfected silkworm larvae at 96 hpi. **(C)** Pattern diagram for measurement of the horizontal distance covered by infected silkworm larvae on a circle grid. **(D)** Quantification by qPCR of the expression of *BmCSP9* in the head of silkworm larvae injected with dsRNA-BmCSP9 or dsRNA-DsRed at 48 and 96 h post injection. **(E)** The locomotion distance of silkworms measured at 96 h after dsRNA injection. **(F)** Violin plots of *BmCSP1* expression in each brain cluster in the BmNPV-infected and uninfected silkworm larvae. **(G)** Quantification of expression of *BmCSP1* by qPCR using the brain samples collected from BmNPV-infected and uninfected silkworm larvae at 96 hpi. **(H)** Quantification by qPCR of the expression of *BmCSP1* in the head of silkworm larvae injected with dsRNA-BmCSP9 or dsRNA-DsRed at 48 and 96 h post injection. **(I)** The locomotion distance of silkworm larvae measured at 96 h after dsRNA injection. Each bar represents the mean ± standard deviation. *p < 0.05, **p < 0.01, ****P < 0.0001. ns, not significant.

As shown in Fig 3A, *BmCSP9* expression was down-regulated by BmNPV infection in the majority of brain cell clusters. Analysis of total brain RNA extracts confirmed that BmNPV infection significantly inhibited the expression of *BmCSP9* in the brain of silkworm larvae at 96 hpi (Fig 3B). Therefore, we speculate that the suppression of *BmCSP9* in the silkworm brain is related to the ELA behavior of the silkworm during late BmNPV infection. Based on previous literature [17,22], we applied a behavioral assay for locomotion activity. The behavior assay evaluates “horizontal” ELA since silkworm larvae are considered too weak for the reliable measurement of climbing behavior [17]. Fig 3C shows a pattern diagram of the dispersion of infected silkworm larvae on a circular grid. When dsRNA-*BmCSP9* was injected into the tail foot of silkworms (in the absence of BmNPV infection), *BmCSP9* expression significantly decreased after 48 and 96 hours in the head (Fig 3D). Nevertheless, the dispersion behavior of the silkworm (as indication of ELA) did not change significantly (Fig 3E), suggesting a different role for this protein in the brain.

Regardless, given the importance of CSPs for the mechanism of olfaction as well as their pleiotropic function in insects [32,34], we also focused on other *CSP* genes such as *BmCSP1* and *BmCSP3* that belong to the top DEGs of the silkworm brain after BmNPV infection (Fig 2A and S1 File). As with *BmCSP9*, *BmCSP1* expression was inhibited by BmNPV infection in various subsets of the silkworm brain at 96 hpi (Fig 3F). In addition, qPCR showed that the expression of *BmCSP1* in the brain of silkworm larvae was significantly inhibited at 96 hpi after BmNPV infection (Fig 3G). As was the case for *BmCSP9*, successful silencing of *BmCSP1* with RNAi after 48 and 96 hours (without virus treatment) (Fig 3H) did not alter ELA of the silkworm larvae (Fig 3I).

### High expression of *BmCSP3* in the brain is an important indication for the induction of ELA by baculovirus infection in silkworm

**Fig 4:**
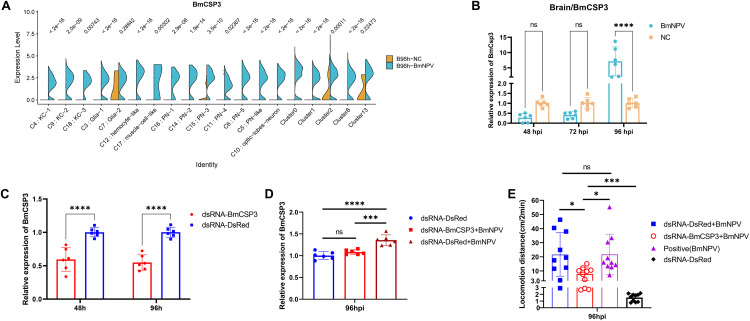
High expression of *BmCSP3* in the brain is a key factor for the induction of ELA behavior during BmNPV infection in silkworm. **(A)** Violin plots of *BmCSP3* expression in each brain cluster in BmNPV-infected and uninfected silkworm larvae. **(B)** Quantification of expression of *BmCSP3* by qPCR using the brain samples collected from BmNPV-infected and uninfected silkworm larvae at 48, 72 and 96 hpi. **(C)** Quantification by qPCR of the expression of *BmCSP3* in the head of silkworm larvae injected with dsRNA-BmCSP3 or dsRNA-DsRed at 48 and 96 h post injection. **(D)** Silencing of expression of *BmCSP3* in the head of silkworm larvae that were co-injected with dsRNA-BmCSP3 and BmNPV-eGFP. The expression of *BmCSP3* in the head was detected by qPCR at 96 hpi. Silkworm larvae co-injected with dsRNA-DsRed and BmNPV-eGFP and non-infected larvae injected with dsRNA-DsRed were used as control. **(E)** The locomotion distance of BmNPV-infected silkworms was measured at 96 hpi for dsRNA-dsRed and dsRNA-BmCSP3 injected silkworms. BmNPV-infected silkworms in the absence of dsRNA injection and uninfected silkworms that were injected with dsRNA-dsRed were used as control. Each bar represents the mean ± standard deviation. *p < 0.05, ***p < 0.001, ****p < 0.0001. ns, not significant.

Another *CSP* gene, *BmCSP3*, is among the highest ranked of all up-regulated DEGs in several subgroups of brain cells (Fig 2A and S1 File). The violin plot also showed that *BmCSP3* expression was upregulated by BmNPV infection in all brain cell clusters (Fig 4A). The induction of expression of *BmCSP3* at 96 hpi was further confirmed by qPCR using whole brain samples (Fig 4B). It is worth noting that the expression of *BmCSP3* in the silkworm brain was not significantly induced by BmNPV infection at 48 and 72 hpi, but instead its expression was inhibited (although not statistically significantly) (Fig 4B). When *BmCSP3* was knocked down using dsRNA-BmCSP3 (without virus treatment), the *BmCSP3* expression significantly decreased after 48 and 96 hours indicating effective knockdown in the head (Fig 4C). Furthermore, the expression of *BmCSP3* remained significantly lower in the head of BmNPV infected and dsRNA-BmCSP3 injected silkworms at 96 hpi (Fig 4D). Interestingly, in BmNPV-infected larvae with knockdown of *BmCSP3*, ELA was significantly inhibited compared to BmNPV-infected larvae that were left untreated or were treated with control dsRNA-Red at 96 hpi (Fig 4E). Also note that compared with uninfected silkworms, ELA behavior is still significantly increased in silkworms infected with BmNPV after knocking down *BmCSP3* (Fig 4E), which can be explained by the incomplete knockdown of *BmCSP3* and/or the involvement of other (not yet identified) host factors. Regardless, these results provide strong evidence that high expression of *BmCSP3* in the brain after BmNPV infection could play an important role in the ELA behavior at the end of the infection cycle.

### Widely targeted lipidomics reveals significant shifts in (lyso)glycerophospholipid abundance during BmNPV infection in silkworm head samples

Because BmCSP3, as a homologue of *Mamestra brassicae* CSPA6 [35], has a large and flexible binding cavity that can accommodate multiple lipid molecules (see further below), it was of interest to determine the lipid content of silkworm tissues in the absence and presence of BmNPV infection for the identification of potential ligands of BmCSP3. Widely targeted lipidomics was performed on head samples that are enriched in nervous (brain) tissue and a comparative analysis was carried out between samples from control larvae and larvae during late infection with BmNPV (96 hpi).

**Fig 5:**
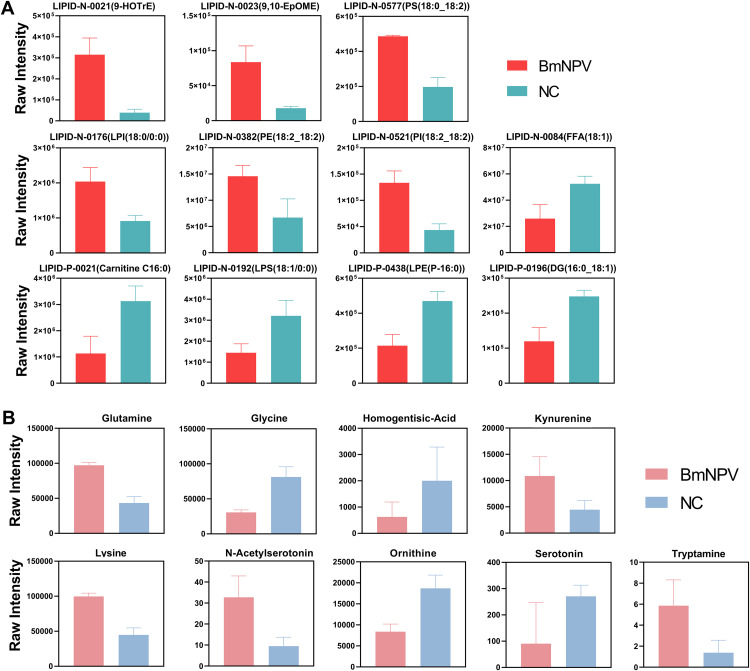
Differential abundance of selected lipids and neurotransmitters between uninfected and BmNPV-infected head samples. **(A)** Quantification of lipids with differential abundance between uninfected and BmNPV-infected head samples. 9-HOTrE and 9,10-EpOME are eicosanoids; PE, PI and PS are glycerophospholipids; LPE, LPI and LPS are lysoglycerophospholipids; DG, diacylglycerol; FFA, free fatty acids. For classification of lipids see [91] and [92]. Detailed information is presented in S2 File. **(B)** Quantification of neurotransmitters with differential abundance between uninfected and BmNPV-infected head samples. Detailed information is presented in S3 File.

Inspection of the 35 lipids that increased in relative abundance during BmNPV infection (S1A and S1B Fig and S2 File) revealed a rather simple pattern that can be summarized as follows. First, there is an increase in all types of glycerophospholipids with different types of two fatty acyl chains with respect to length and double bond position. Second, within the three top up-regulated lipids, two were characterized as eicosanoids: (10E,12Z,15Z)-9-hydroxyoctadeca-10,12,15-trienoic acid (9-HOTrE; log2FC = 3.02) and 9,10-epoxy-12Z-octadecenoic acid (9,10-EpOME; log2FC = 2.24) (Fig 5A and S2 File).

Inspection of the 147 lipids that were down-regulated (S1A and S1B Fig and S2 File) indicates a strong decrease in triglycerides which, concomitant with the increase in glycerophospholipids, can be interpreted as a switch in function from energy storage to membrane formation [36]. There is also a decrease in lysoglycerophospholipids (also called monoacylglycerophospholipids) while (diacyl) glycerophospholipids increase. Interestingly, lysoglycerophospholipids have a much bigger role as signaling molecules in the brain [37]. Ceramides, another class of lipids with signaling in addition to structural function [38], are also decreased in abundance (S2 File). Finally, a decrease in carnitine, an important regulator of fatty acid oxidation in mitochondria [39], is detected.

The major change in glycerophospholipid metabolism after BmNPV infection was confirmed by KEGG enrichment analysis of differential lipids (S1C and S1D Fig). Changes in abundance of selected lipids (used for docking analysis, see further below) in BmNPV-infected silkworm head are presented in Fig 5A.

### Changes in abundance of neurotransmitters following BmNPV infection

The high expression of *BmCSP3* in the brain and its strong regulation by BmNPV infection raised the question whether BmCSP3 could bind to neurotransmitters and whether such binding could be correlated with the differential abundance of neurotransmitters that might occur during BmNPV infection. Targeted metabolomics was performed to quantify the changes in abundance of a group of 55 neurotransmitters and possible precursors and metabolites following BmNPV infection (96 hpi). Differential metabolite screening, based on FC ≥ 2 or FC ≤ 0.5, identified nine substances of which five have a P-value lower than 0.05 (S3 File). Fig 5B shows the violin plots that illustrate the differential abundance of the nine neurotransmitter-related molecules with │log2FC│ ≥ 1. The differential metabolites are related to the metabolism of amino acids, most notably tryptophan (involving kynurenine (up), tryptamine (up), serotonin (down) and N-acetyl-serotonin (up) (Fig 5B) [40,41].

## Modeling of the BmCSP3 structure

To obtain more information about the role of this chemosensory protein and to deduce its possible ligand specificity, we determined three-dimensional homology models of BmCSP3. A search for structural templates at SWISS-MODEL [42] using the sequence of the mature BmCSP3 (UniProt ID: Q3LBA2, aa 19–127) revealed four CSPs with sequence identities of 52–54% (S4 File). In particular, the structure of BmCSP1, which becomes down-regulated following BmNPV infection (Fig 3), has been resolved by Nuclear Magnetic Resonance (NMR) [43] and revealed a similar structure to the X-ray crystal structure of CSPA6 from *Mamestra brassicae* (CSPMbraA6; also called CSP2) [44].

**Fig 6:**
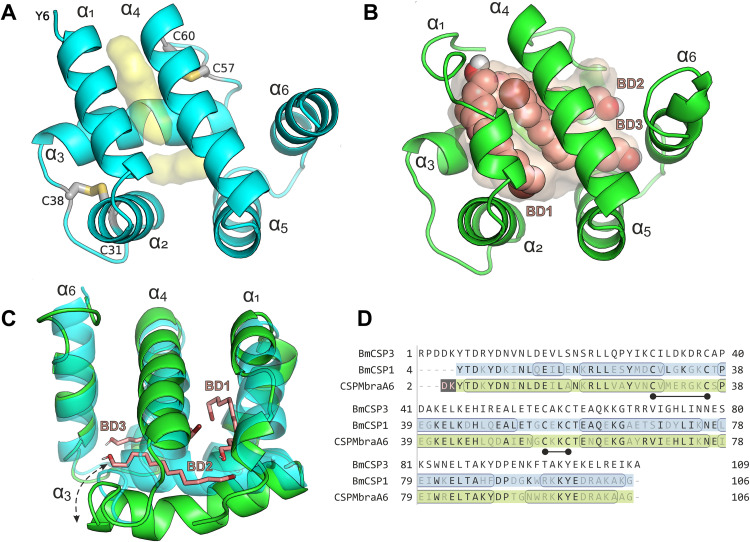
Molecular modeling of BmCSP3 based on CSPMbraA6. **(A)** Homology model of BmCSP3 in the ligand-free (closed) state. The internal cavity is shown as transparent yellow surface and the 6 helices are designated α1–α6. The two conserved disulfide bridges are shown with their side-chain atoms in sticks and the corresponding cysteine residues are labelled. **(B)** Homology model of BmCSP3 in a ligand-bound state (open) with three molecules of 12-bromo-1-dodecanol (BD1–BD3). The ligands are shown with spheres (pink C atoms, red O and brown Br) and are positioned according to the template structure of CSPMbraA6 (PDB ID: 1n8v). **(C)** Superposition of the two modeled (closed and open) states of BmCSP3 shown from the opposite side of the cavity to exhibit the large conformational shift of α3 indicated by the dashed arrow. **(D)** Sequence alignment of BmCSP3, BmCSP1 and CSPMbraA6 with identical residues highlighted. Residues that comprise the 6 α-helices are circled (according to PDB IDs: 2jnt for BmorCSP1; 1kx9 for CSPMbraA6), whereas the conserved cysteine residues that form disulfide bridges are indicated with the black nodes. Residues in pink on brown were not well-defined for structural determination.

Interestingly, CSPMbraA6 has been determined in both a ligand-free (closed) conformation and a ligand-bound (open) form that displayed a much larger internal cavity and therefore provides two interesting templates for the homology modeling of BmCSP3. Taking into account the equally high sequence identity and coverage of BmCSP3 with both BmCSP1 and CSPMbraA6 (Fig 6D), we employed CSPMbraA6 in the ligand-free, closed form (PDB ID: 1kx9) [44] and the ligand-bound, open form (PDB ID: 1n8v) [35] as templates for the generation of BmCSP3 homology models (Fig 6A, 6B, 6C and S2 Fig). This decision implies the potential that BmCSP3 may also undergo large conformational changes to accommodate long and bulky ligands similar to CSPMbraA6, a hypothesis which can also be valid for BmCSP1, in particular considering that the last two share 70% sequence identity [43].

The model of BmCSP3 in the closed, ligand-free state reveals a hydrophobic cavity formed by the pairs of helices α1-α2 and α4-α5, which are arranged in the two V-shaped faces of the cavity and are closed by the face of helix α3 in a prism-like fashion (Fig 6A). The amphiphilic helices are comprised mainly of leucine, isoleucine and valine residues at their hydrophobic side of the cavity that is discontinued by a conserved tyrosine residue (Y28 in BmCSP3, Y26 in BmCSP1 and CSPMbraA6) (Fig 6D). The side-chain of this residue can also adopt a conformation that allows extension of the narrow and elongated cavity (S3 Fig; compare chain A and chain B which represent the alternative conformations of the closed form), so that even long acyl-chain lipid molecules (C12-C18 atoms) can be accommodated [44].

Investigation of the ligand-bound (open) form of the BmCSP3 model (Fig 6B) reveals a very similar architecture of the larger cavity with that of the template structure of CSPMbraA6 [35]. Therefore, the three bound 12-bromo-1-dodecanol molecules that were resolved in its X-ray structure could be retained in the model of BmCSP3 with only a few minor short contacts. The interacting residues at this extended binding site are comprised of 20 identical and seven similar residues (mainly swaps among valine, leucine and isoleucine), and only two non-conservative substitutions (Gly ↔ Glu and Arg ↔ Thr; S4 Fig). It should be noted that the latter side-chain changes do not affect ligand binding directly but probably offer a distinct role in each CSP. Comparison of the two main openings found in the CSPMBraA6 cavity towards the bulk solvent, which are surrounded by residues Ile51, Trp94, Tyr98 and by Leu13 and Asn61, respectively, [35] reveals that the corresponding residues of BmCSP3 are quite similar (Leu53, Phe96, Tyr100 for the first, and Leu15 and Ala63 for the second), so that even long-chain aliphatic molecules can extend with their polar moiety towards the solvent. A smaller opening detected between Tyr4, Glu42 and His46 of CSPMbraA6 is very similar to BmCSP3 comprising the identical residues Tyr6, Glu44 and His48. Taken together, the similarities of the ligand-free (closed) and lipid-bound (open) models of BmCSP3 with the corresponding structures of CSPMbraA6 suggest that these CSPs may share properties of extracting and transporting hydrophobic, long linear and aliphatic compounds that reside in membranes.

### Molecular dynamics study of BmCSP3

**Fig 7:**
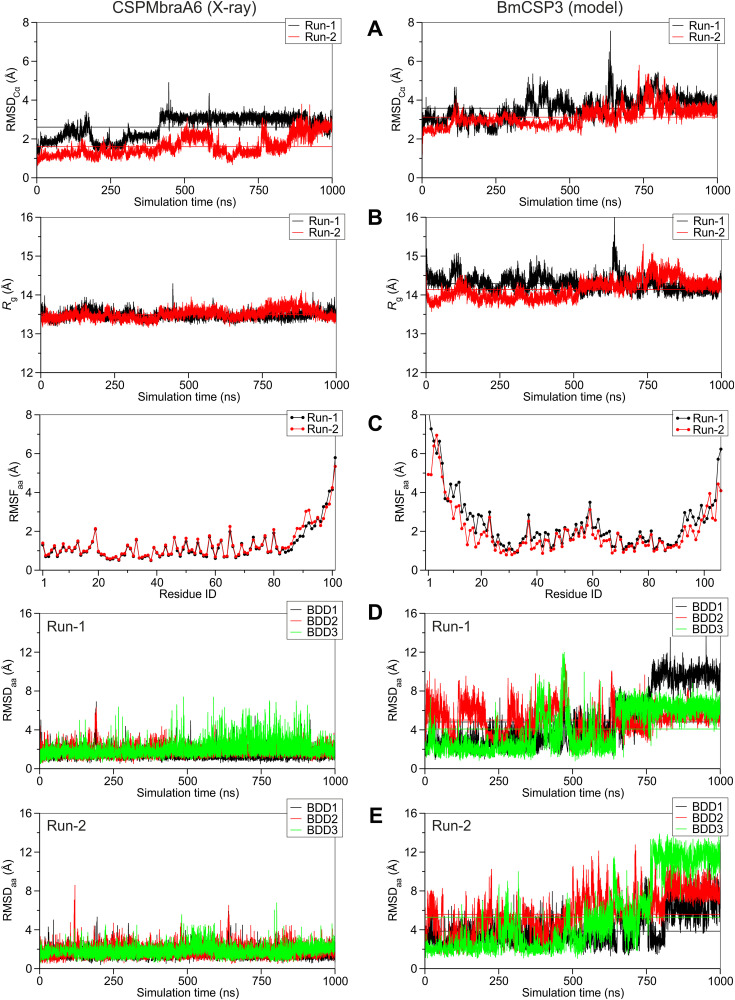
Molecular dynamics study of BmCSP3 compared to CSPMbraA6. Plots of geometric features extracted from two independent 1-μs timescale MD simulations (designated as Run-1 and Run-2) using the X-ray structure of CSPMbraA6 (PDB ID: 1n9v, left panels) and the homology model of BmCSP3 (right panels). **(A)** Root-mean-square deviation of the Cα backbone atoms (RMSD_Cα_) as a function of simulation time for the two proteins, indicating changes in the main-chain from the initial position. **(B)** Radius of gyration (*R*g) as a function of simulation time reveals the distribution of the protein atoms around its axis. **(C)** Root-mean-square fluctuations of all heavy atoms (RMSF_aa_) showing average atomic fluctuations per residue over the total simulation time. **(D-E)** RMSD of all heavy atoms for the 3 bound ligands (BDD1–BDD3 in the order appearing in PDB ID 1n9v) from their initial position as a function of simulation time in the two MD runs.

To investigate further the lipid-bound form of the BmCSP3 model in its more open form, we carried out molecular dynamics simulations (MDs) in explicit solvent. Two simulations were initiated at 25ºC for each of the lipid-bound forms of BmCSP3 and CSPMbraA6 for comparison, which were extended up to 1 microsecond simulation time without any restraints. Our results indicate that the bound lipids are significantly more flexible in BmCSP3 than in CSPMbraA6, however still not that mobile as to dissociate within this timescale. This difference may be attributed to the overall higher flexibility of the BmCSP3 homology model compared to CSPMbraA6 [35]. The apparent higher flexibility of the BmCSP3 model exhibits higher backbone displacement from the starting structure (RMSD_Cα_ in Fig 7A) and displays even more open conformations than its template structure of CSPMbraA6 (*R*_g_ in Fig 7B). This observation can be attributed to the significantly higher fluctuations displayed by the N-terminal helix α1 and part of helix α2 in BmCSP3 compared to CSPMbraA6 (RMSF in Fig 7C), thus giving rise to a larger and more solvent accessible cavity for the ligands. As a result, the three 12-bromo-1-dodecanol ligands (BDD1-BDD3 in Fig 7D and 7E) show significantly higher mobility and positional shifts from their initial bound pose in BmCSP3 compared to CSPMbraA6. Taken together, this analysis indicates that BmCSP3 may adopt even more open and solvent accessible forms than CSPMbraA6, or that the ligand-bound model of BmCSP3 complex with three 12-bromo-1-dodecanol molecules is not a very stable state of this protein.

### Molecular docking of selected lipids and neurotransmitters in the BmCSP3 models

**Fig 8:**
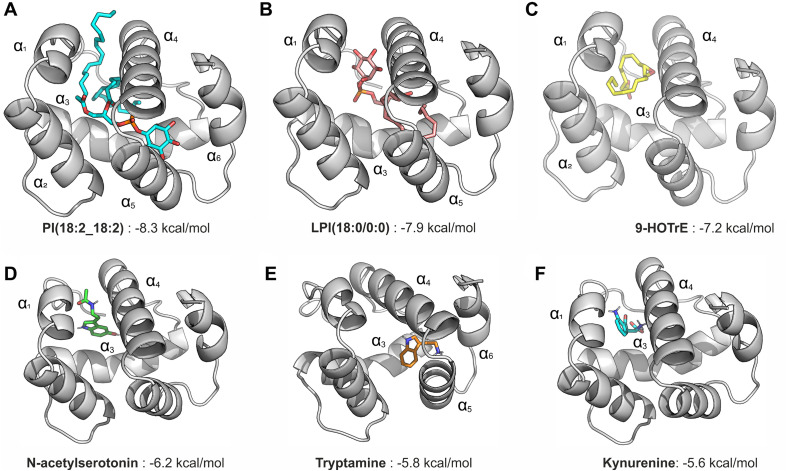
Models of ligand-bound BmCSP3 in the open state. Models of ligand-bound BmCSP3 in the open state, illustrating the lowest energy docked pose and the corresponding score (predicted binding free energy in kcal/mol) for: **(A)** phosphatidylinositol PI(18:2_18:2); **(B)** lysophosphatidylinositol LPI(18:2/0:0); **(C)** 9-HOTrE; **(D)** N-acetylserotonin; **(E)** tryptamine; **(F)** kynurenine. The 6 helices that define the protein structure are labeled as α1–α6.

With the aim of predicting structural models of ligand-bound BmCSP3, we employed the nine neurotransmitter-related compounds and a selection of 15 lipids that displayed │log2FC│ ≥ 1 (six up-regulated and nine down-regulated). Docking calculations were carried out using the BmCSP3 models in the closed state and in the open state. Our results indicate that the lipids employed cannot fit inside the binding groove of BmCSP3 in the closed state (S5A Fig), which is also the case for the bulkier neurotransmitter-related compounds (see for example kynurenine and N-acetylserotonin in S6A Fig). In contrast, the open conformation of CSP3 can accommodate all lipids and neurotransmitter-related compounds, an observation that is also reflected in the corresponding binding scores (S5B and S6B Figs). The bulkier C18-phosphatidylinositol (PI (18:2_18:2)) displayed the highest binding affinities, followed by its lyso-counterpart C18-lysophosphatidylinositol (LPI (18:0/0:0)) and the eicosanoid 9-HOTrE (Fig 8A-C). Regarding the neurotransmitter-related compounds N-acetylserotonin, tryptamine and kynurenine (Fig 8D-8F) were predicted to bind stronger than the amino-acids glutamine, lysine, ornithine and glycine (S6C Fig).

### Binding affinity of neurotransmitters and lipids

**Fig 9:**
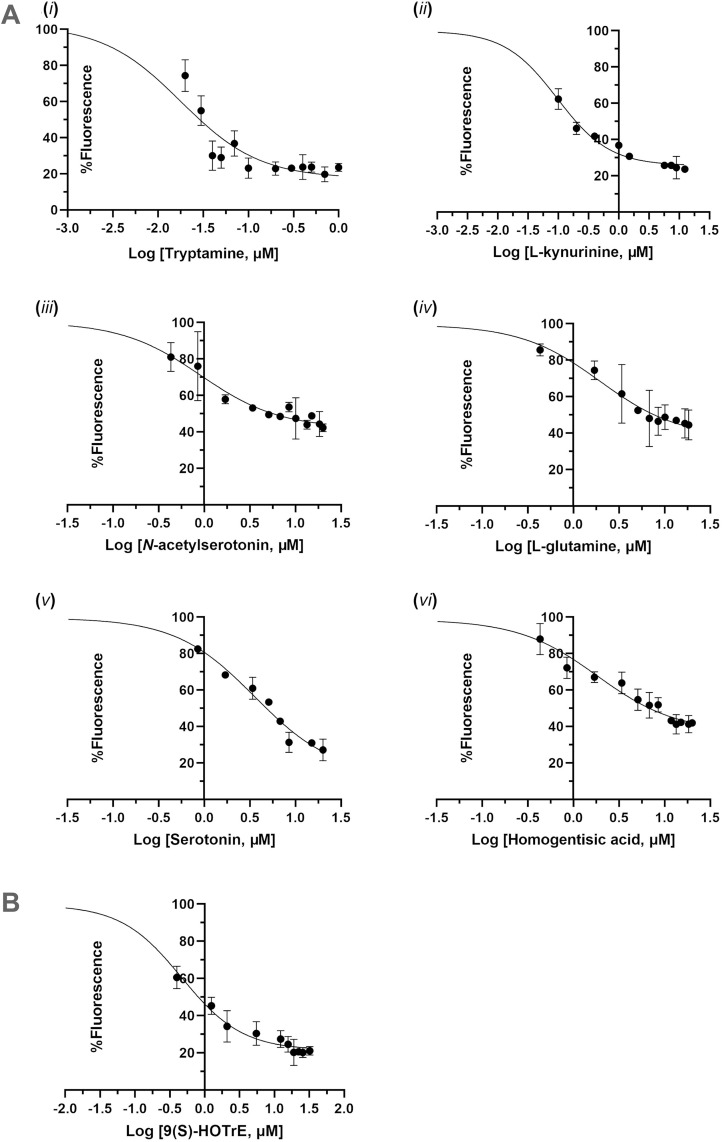
Binding of neurotransmitters and lipid to BmCSP3 evaluated by fluorescence displacement binding assays. **(A)** Dose-response curves illustrate the effect of the tested neurotransmitters (Table 1) on the displacement of the fluorescent probe 1-NPN from BmCSP3. Fluorescence measurements were conducted using 4 μM BmCSP3 and a constant concentration of 1-NPN (10 μM), with neurotransmitter concentrations ranging from 0 to 20 μM. The response was expressed as the percentage reduction in fluorescence relative to the fluorescence observed in the absence of ligand (defined as 100%). Fluorescence data were plotted against the logarithm of ligand concentration (μM). **(B)** Fluorescence measurements were conducted using 4 μM BmCSP3 and a constant concentration of 1-NPN (10 μM), with 9(S)-HOTrE concentration ranging from 0 to 32 μM.

As shown in Table 1 and Fig 9A, all tested neurotransmitters, except for the amino acids lysine, glycine, and ornithine, were identified as BmCSP3 binders. Among these, tryptamine and kynurenine were found to be strong binders, with *K*i values in the nanomolar range (11 nM and 72 nM, respectively). These were followed, in order, by serotonin, *N*-acetylserotonin, homogentisic acid, and glutamine, which showed Ki values ranging from 1.8 to 2.8 μM. Among the lipids, 9(S)-HOTrE, the most elevated metabolite in the BmNPV-infected state, exhibited strong binding, with a *K*i value of 290 nM (Table 1 and Fig 9B).

**Table 1:** *In silico* and *in vitro* binding of the tested neurotransmitters/lipids.

Neurotransmiter	Fold Change	Docking Score to open form (kcal/mol)	Maximum fluorescence decrease % (at ligand concentration)	EC_50,_*int* ± SE (μΜ)	*K*i (μΜ)
Tryptamine	4.26	-6.5	76.5% (1 μΜ)	0.03 ± 0.01	0.011
*N*-acetylserotonin	3.45	-7.3	57.7% (20 μΜ)	5.82 ± 1.13	2.13
L-kynurenine	2.44	-6.5	76.4% (12 μΜ)	0.20 ± 0.02	0.072
L-glutamine	2.24	-4.8	55.6% (18 μΜ)	7.78 ± 1.48	2.8
L-lysine	2.23	-4.8	0.0% (14 μΜ)	Ν/Α*	Ν/Α
Homogentisic acid	0.31	-6.1	58.1% (20 μΜ)	6.76 ± 1.58	2.5
Serotonin	0.33	-6.6	72.9% (20 μΜ)	4.81 ± 1.17	1.8
Glycine	0.38	-3.3	9.7% (20 μΜ)	Ν/Α	Ν/Α
DL-Ornithine	0.45	-4.4	0.0% (20 μΜ)	Ν/Α	Ν/Α
**Lipid**					
9(S)-HOTrE	8.10	-7.2	78.9% (32 μΜ)	0.79 ± 0.10	0.29
(±)9(10)-EpOME	4.71	-7.0	11.9% (0.1 μΜ)	ND**	ND

*Maximum fluorescence decrease <50%

**Interference with 1-NPN

%Fluorescence Intensity (%FI) represents the percentage of fluorescence intensity measured at the maximum ligand concentration used in the assay. EC₅₀ is the effective concentration required to cause a 50% decrease in fluorescence (50% displacement of 1-NPN). *Ki is t*he dissociation constant value of the ligand, calculated using the Cheng-Prusoff equation under competitive conditions (10 μM 1-NPN, *Kd*^*1-NPN*^ = 5.8 μM). Fold change in relative abundance of neurotransmitters/lipids in the BmNPV-infected group and their docking scores to CSP3 “Open” (*in silico*) are also presented.

## Discussion

BmNPV is a common silkworm pathogen that also infects the central nervous system (CNS) where it induces ELA behavior [22]. Because silkworm behavior is coordinated by the brain, the question arises about the mechanism by which BmNPV affects the host brain functions to elicit ELA. Identifying the specific cell types in the brain targeted by the virus is crucial for understanding the pathogenesis of BmNPV infection and the host behavior controlled by BmNPV infection. We have previously reported a comprehensive single-cell atlas of the silkworm brain during BmNPV infection [11]. Nineteen transcriptionally distinct cell subtypes in the silkworm brain were identified, including three Kenyon cell (KC), two glia cell, six olfactory projection neuron (PN), one optic lobe neuron, one hemocyte-like, one muscle cell-like and five unannotated clusters [11].

In this work, we show that at the late stages (96 hpi) of BmNPV infection all cells in each brain cell cluster are virally infected, although the viral load in infected cells is generally low (except for cluster 2) (Fig 1). For viral factors, it was reported that the group I NPV *ptp* gene plays a key role in inducing hyperactive behaviors [14,15,24]. Interestingly, we found that the *ptp* gene is not expressed in all brain cells, but mainly in cluster 2 cells (Fig 1G). Coincidentally, the number of cluster 2 cells in the brain of silkworms infected with BmNPV significantly increased compared to non-infected silkworms (by 61-fold, up to 13% of all brain cells) [11]. While PTP was recently identified as a structural component of the virions of group I alphabaculoviruses [26], its phosphatase activity was not required for ELA activity in BmNPV, in contrast to AcMNPV [24]. The high expression of *ptp* in cluster 2 may reflect the high production of virions in the brain that contain PTP in the virion structure. Additionally, a total of 36 proteins have been identified as potentially interacting with PTP of BmNPV in the silkworm brain [23]. However, among the 61 DEGs identified in cluster 2 after BmNPV infection at 96 hpi (S1 File), no genes encoding the putative PTP interaction proteins were found. How this observation may relate to changes in brain function (induction of ELA) is not clear and will require the elucidation of the function of the cells of cluster 2. Unfortunately, the exact identity of cluster 2 could not be defined based on marker genes from the literature. Regardless, the identification of a specific cell cluster in the brain that is more sensitive to BmNPV infection and is capable of sustaining *ptp* expression is intriguing and deserves more attention in future experiments.

We have found clues about how BmNPV could induce ELA in silkworm larvae by investigating the changes in the expression of the host’s CSPs. In the present study, we identified six subsets of olfactory projection neurons (PN1 to PN5 and PN-like), but only one subset of optic lobe neurons. The proportion of PN and PN-like neurons increases from 3% to 16% during BmNPV infection, while olfactory lobe neurons remain relatively low (increase to 2.5%) [11]. Moreover, expression of several *CSP* genes encoding olfaction-related proteins was found to be significantly altered in brain clusters after infection with BmNPV (Fig 2A). CSPs were originally identified by their involvement in peripheral olfactory processing and are considered as one of the most important classes of proteins for chemoreception due to their functions as carriers of semiochemicals [32]. Chemoperception of caterpillars can indeed be altered during baculovirus infection through changes in the expression level of specific odorant receptors [45].

Several mechanisms can be proposed by which changes in CSP expression during baculovirus infection could trigger ELA behavior. One hypothesis would assume that the changes in *CSP* expression disrupt the normal function of the olfactory system, leading to ELA behavior. However, the typical changes in *CSP* expression during infection (upregulation of *BmCSP3*, downregulation of *BmCSP1* and *BmCSP9*) are not restricted to PN (-like) clusters but occur in many of the clusters, including the abundant (undesignated) clusters 0, 1 and 2 (Figs 2-4 and S1 File). For their role in olfaction, CSP proteins are not expected to be expressed in neurons but in the cells of olfactory sensilla in the antennae (absent in larvae) [46]. On the other hand, additional roles of CSP proteins have been proposed recently, including functions in development and regeneration [34]. Accordingly, it can also be hypothesized that the shift in *CSP* expression that occurs in the majority of brain cells underlies fundamental changes in CNS structure and function, which affects silkworm behavior and leads to ELA. The challenge for future studies would be to elucidate the specific mechanism of ELA mediated by BmCSP3 in BmNPV-infected silkworm. It is worth noting that knocking down *BmCSP3* did not completely eliminate BmNPV-induced ELA (Fig 4E). Possible reasons include 1) the retained BmCSP3 was still functioning; 2) there were other host factors in the silkworm brain that could affect BmNPV-induced ELA.

CSPs are evolutionary conserved proteins, and generally function as carriers of hydrophobic ligands [44]. The number of available structures of CSPs is relatively low (S4 File) and the most comprehensive studies were carried out with CSPMbraA6 of the moth *M. brassicae* [35,44,47]. Homology modeling of BmCSP3 (Fig 6) revealed very similar structural features to CSPMbraA6, both in ligand-free (closed) and ligand-bound (open) forms, probably because of their relatively high sequence identity (54%). More specifically, BmCSP3 and probably the closely related BmCSP1 contain a ligand-binding pocket that can undergo large conformational changes so that it can accommodate multiple copies of long-chain ligands such as dodecanol or yet unknown more complex, branched or bulky, ligands of physiological relevance. In the X-ray crystal structure of the CSPMBraA6 homologue, three elongated 12-bromo-dodecanol molecules were identified in a wide and solvent accessible cavity [35]. The BmCSP3 model may share a similarly large and flexible hydrophobic pocket with minor differences in the binding of the three ligands (Fig 6). Further investigation of the ligand-bound form of BmCSP3 with MD simulations displayed an even more flexible protein–ligand complex compared to CSPMbraA6, especially at the N-terminal α1–α2 helices (Fig 7). This may reflect that BmCSP3 has different specificity than CSPMbraA6 and may indicate its property to accommodate complex or multiple ligands, as indicated from the literature.

In binding experiments with CSPMBraA6, positive cooperativity and apparent dissociation constants of 0.19-1.6 μM were observed for a series of alkyl compounds [35,48]. Binding was not affected much by length, saturation or chemical function. Long alkyl chains were proposed to extend in the hydrophobic cavity while chemical groups (acetate, alcohol, acid, aldehyde) could point out in the bulk solvent [35]. By contrast, bulky compounds hardly could bind. With respect to *S. litura* CSP8, besides binding of the long linear molecules bombykol and 12-bromododecanol, relative high affinity was also detected for rhodojaponin III, a more rigid plant-derived compound comprising of the complex ring-system of diterpenoids [49]. Binding to the amide derived from the omega-9 fatty acid oleic acid was observed for CSPsg4 from the desert locust [50]. Thus, molecules with long alkyl chains, such as (saturated and unsaturated) fatty acids and their metabolites, can be considered as ligands for CSPs expressed in the brain.

Recently, high-throughput lipidomics studies were applied to silkworms [51–53]. In the present work, to identify relevant ligands for BmCSP3 in the brain, widely targeted lipidomics was performed using head samples of larvae that were either uninfected or infected with BmNPV for 4 days (Figs 5A and S1 and S2 File). The analysis of differential lipids showed a shift from triglycerides to glycerophospholipids during BmNPV infection, which was also previously observed for whole bodies of silkworms [51]. A recent study confirmed a role for lipid metabolism and its regulation by adipokinetic hormones during infection of Helicoverpa armigera NPV, which affected climbing behavior [54]. In the present study, unexpectedly, two of the three lipids with the highest increase in abundance included two eicosanoids that have known signaling properties [55,56]. Targeted metabolomics was also performed and identified 9 candidate neurotransmitters with differential abundance (Fig 5B and S3 File). Docking studies showed that all tested differential lipids and neurotransmitters could bind with relatively high affinity to the open conformation of BmCSP3 (S4 and S5 Figs), which reflects its broad binding capacity and affinity for hydrophobic molecules. Since *BmCSP3* expression increases during BmNPV infection at 96 hpi (Fig 4), it makes sense to hypothesize its involvement in the binding of up-regulated metabolites. The detected lipids have high abundance (see values in bar plots; Fig 5A) and may therefore be more readily available for interaction with BmCSP3. On the other hand, among neurotransmitters, kynurenine, glutamine and lysine are present at sufficiently high post infection levels (Fig 5B), while the low abundance of others may reflect their presence in only a few cells of the brain. Increased ligand availability in the disease state could enhance the formation of BmCSP3-ligand complexes, particularly for those with high binding affinity, as suggested by docking calculations against BmCSP3 “open” (Fig 8D-F) and confirmed by *in vitro* assays (Fig 9 and Table 1). These combined results support the notion that these ligands (high affinity-(local) high abundance) may represent physiologically relevant interactors of BmCSP3 in the ELA state. The effect of ligand binding to the equilibrium between open and closed BmCSP3 states is beyond the scope of this work. However, future crystallographic analyses may support the proposed ligand-induced mechanism that underlies transition between these two forms.

In the CSPMbraA6 open structure, three elongated 12-bromo-dodecanol molecules were identified, which raises the question whether BmCSP3 can also encompass multiple ligands. Our docking calculations indicate that glycerophospholipids, which are much bulkier, can be accommodated only as a single ligand inside the binding cavity of BmCSP3 (Fig 8A). In contrast, eicosanoids and the much smaller neurotransmitters may occupy multiple sites at the same time (Fig 8C-F).

*In vitro* binding assays with purified BmCSP3 protein were performed to confirm the interaction with the neurotransmitters that changed in abundance during BmNPV infection (Table 1 and Fig 9A). Especially tryptamine and kynurenine show high affinity for BmCSP3 binding. Interestingly, among the metabolites with differential abundance, tryptamine, kynurenine, and serotonin are all metabolites of tryptophan, produced via distinct metabolic pathways, that compete for the available tryptophan. A recent study demonstrated that tryptophan levels decreased markedly 120 hours post-BmNPV infection in susceptible silkworm strains [57]. Furthermore, exogenous supplementation of tryptophan was found to inhibit BmNPV replication, underscoring the significant role of tryptophan and its related metabolic pathways in viral infection and host metabolic reprogramming [57]. In our study of metabolic programming during BmNPV infection, however, the levels of tryptophan increase in the hemolymph of BmNPV-infected larvae at 72 hpi [58]. Therefore, the upregulation of BmCSP3 and its affinity for critical neuromodulators provide direct evidence of its contribution to the behavioral changes observed during baculovirus infection, possibly by affecting the balance among tryptophan metabolic pathways. Nevertheless, further studies are necessary to elucidate the specific mechanisms by which CSP3 influences behavior during baculovirus infection.

In addition to small molecule neurotransmitters, the binding to BmCSP3 of 9(S)-HOTrE, the top lipid metabolite with the highest increase during BmNPV infection, was validated in binding assays (Table 1 and Fig 9B). In contrast, it was not possible to determine the *K*i value for (±)9(10)-EpOME. In control samples lacking protein, a concentration-dependent increase in 1-NPN fluorescence was observed in the presence of (±)9(10)-EpOME, which was further pronounced in the presence of protein (S7 Fig), suggesting that EpOME may form micelles whose hydrophobic environment can artificially enhance the 1-NPN signal. A similar ligand-induced effect has been previously reported for compounds such as the mosquito oviposition pheromone, (Z)-16-hexadecenyl acetate, and the alarm pheromone (E)-β-farnesene [59–61]. Nevertheless, based on the observed reduction in fluorescence (11.9%) at the lowest tested concentration (0.1 μΜ), we anticipate that (±)9(10)-EpOME is also a BmCSP3 binder. Its *K*d value remains to be determined in future studies employing an alternative fluorescent probe or by a different binding assay, such as isothermal titration calorimetry. While the focus was on the highly ranked eicosanoids that have documented signaling properties, binding of other lipids (e.g., (lyso)glycerolipids) can also be expected in the *in vitro* assays, especially to CSP3 in its open conformation, conform to our modeling studies (S5 Fig).

The binding of lipids and neurotransmitters to BmCSP3 can have both positive and negative effects, which need to be verified experimentally. Sequestration or transport of lipids or neurotransmitters by CSPs could affect the function of neurons in the brain directly but also indirectly, such as changes in membrane fluidity and the availability of second messengers or cytokine precursors [62]. In mammalian neuronal tissue, lipids can interact with both membrane and nuclear receptors to regulate physiological and pathological processes [63,64]. Poly-unsaturated fatty acids are known for their role in synapse function in both vertebrates and invertebrates [65], while saturated fatty acids affect memory formation in rats [66]. In the brain, CSPs could therefore act as lipid-binding proteins that regulate neuron differentiation and function [67]. Furthermore, long-chain N-acylserotonin and N-acyldopamine production was demonstrated in *D. melanogaster* [68], which connects fatty acids with monoamine neurotransmitters. When considering the results of differential lipidomics of the head samples, a large shift in signaling pathways can be considered where eicosanoids are increased and lysophospholipids and ceramides are decreased [37,38]. Strikingly, two of the lipids with the highest change in abundance are eicosanoids that act as signaling molecules in other experimental systems: 9-HOTrE is an oxylipin with immune-modulating effects [55,69] and 9,10-EpOME was designated as a leukotoxin because of its negative effects on mitochondrial respiration [56]. Glycerophospholipids can be precursors of eicosanoids [70,71] and the increased abundance of glycerophospholipids may be related to the production of eicosanoids. However, the shift from triglycerides to phospholipids has also been related to the immune response and may be involved with the increased secretion of immune effectors [72].

In conclusion, our snRNA-seq data provide a resource for a comprehensive system-level understanding of the silkworm brain and its response to viral infection. All brain cells could be infected by BmNPV at 96 hpi. More importantly, we found that high expression of BmCPS3 in the brain led to the emergence of ELA behavior. While CSPs were found previously to regulate behavior by their sensory functions (e.g., [60,73]), our research also points to a more direct role of CSPs and especially of CSP3 in the extracellular milieu of the brain tissue. A major research direction would be to attempt to reconcile the results of the differential metabolomics with the differential expression of CSPs in the brain during BmNPV infection, i.e., by the investigation of their role as ligands of CSPs in nervous tissue. Such studies can establish specific CSPs as molecular targets involved in insect brain function and pave the way for biotechnological applications to manipulate insect behavior.

## Materials and methods

### Silkworm and virus infection

Larvae of silkworm (Dazao strain) were reared with fresh mulberry leaves and reared at a temperature of 28°C and humidity between 60 and 70%. Recombinant BmNPV-eGFP (Enhanced Green Fluorescent Protein), as a reporter virus (budded virus phenotype), was constructed by the BmNPV-based Bac to Bac System (*Bombyx mori* MultiBac) [74]. Newly molted fifth-instar silkworm larvae were injected with either 10 μL of BmNPV-eGFP (1 × 10^6.2^ TCID_50_/mL) or phosphate buffered saline (PBS) (Negative Control, NC).

### Detection of BmNPV and *CSP* genes expression in the brain of infected silkworms

Brain tissue sample was collected from pools of six BmNPV-infected (B96h-BmNPV) and six control (B96h-NC) silkworm larvae under the stereomicroscope. Three duplicate samples were collected in each experiment for the infection and control group. The brain tissue was cleaned with DEPC water, flash frozen and stored in liquid nitrogen until further use.

Total RNA of brain tissue was extracted by Kit RNA fast 2000 (Fastagen, Shanghai, China) and reverse transcribed to cDNA by a RT reagent kit with gDNA Eraser (TaKaRa, Shiga, Japan). The viral gene *vp39* was used to detect viral mRNA abundance, and the silkworm *TIF4A* gene was used as an internal control. All primer sequences are listed in S5 File.

Brain protein samples were prepared by RIPA lysis buffer (Beyotime), separated by SDS-PAGE electrophoresis and transferred to polyvinylidenefluoride membranes for incubation with mouse anti-GFP (1:2000 dilution) or ɑ-tubulin antibodies (1:2000 dilution). Horseradish peroxidase-conjugated goat anti-mouse (Beyotime) (1:2000 dilution) was used as secondary antibody. Western blot signals were detected with the ECL Western Blotting Detection System (Bio-Rad).

qPCR for detection of *CSP* mRNA was performed on the Bio-Rad CFX96 Real-Time Detection System using iTaq Universal SYBR Green Supermix Kit reagents (Bio-Rad, Hercules, CA, USA). The silkworm *TIF4A* gene was used as an internal control. The primer sequences of silkworm *CSP* genes including *BmCSP9* (BMSK0011182), *BmCSP1* (BMSK0011156) and *BmCSP3* (BMSK0011153) are listed in S5 File.

### Viral gene expression analysis in cell clusters of the BmNPV-infected brain

The samples, snRNA seq raw data, and data processing methods used in this study are the same as those used in our published study [11]. At 96 hpi of BmNPV-eGFP, brain tissue was collected from pools of twenty BmNPV-infected silkworms and twenty control larvae. Each of the two experimental groups was collected as a pooled sample that was respectively named B96h-BmNPV and B96h-NC.

To determine the state of BmNPV infection in each silkworm brain cluster, the intranuclear virus mRNA was analyzed in the different cell clusters in the BmNPV-infected group. Viral gene expression was analyzed using Cell Ranger based on the BmNPV genome (GenBank: JQ991008.1). The ‘viral load’ of a cell in snRNA-seq analysis was based on the number of Unique Molecular Identifiers (UMIs) that map to the BmNPV genome and expressed as a percentage of the total UMI (UMI BmNPV + UMI host) content of a given cell [75].

### Knockdown of *CSP* genes in silkworm larvae

Double-strand RNAs (dsRNAs) specifically targeting *BmCSP9* (BMSK0011182), *BmCSP1* (BMSK0011156) and Bm*CSP3* (BMSK0011153) mRNAs were designed and used in knockdown experiments. The primers for dsRNA synthesis are listed in S5 File. T7 RiboMAX Express RNAi System (Promega, USA) was used to synthesize dsRNA, and the products were stored at −80°C until use. DsRNA-DsRed was used as control. Silkworm larvae (5th instar) were injected with 5 μL of dsRNA (6 μg) into the tail foot. The knockdown effect was detected by qPCR using head samples from 3-5 animals selected randomly from all treated silkworms at 48 and 96 h post injection of dsRNA. In addition, 3–5 silkworms injected with dsRNA-CSP3 and BmNPV were randomly selected for *BmCSP3* expression detection at 96 hpi. qPCR was performed on the Bio-Rad CFX96 Real-Time Detection System using iTaq Universal SYBR Green Supermix Kit reagents (Bio-Rad, USA). The silkworm *rp49* gene was used as an internal control. All primer sequences are listed in S5 File. Data analyses were performed using the 2^-ΔΔCt^ method.

Additionally, to investigate the effect of *CSP* genes on ELA behavior, silkworm larvae (5th instar) were respectively injected with dsRNA-CSP9, dsRNA-CSP1*,* dsRNA-CSP3 and dsRNA-DsRed (control) into the tail foot. Because the expression of *BmCSP9* and *BmCSP1* is inhibited by BmNPV infection in the brain (according to the snRNA-seq results), the knockdown silkworms were analyzed in the absence of virus infection for measurement of ELA behavior at 96 hours after target gene knockdown. In the case of *CSP3* expression, which is induced by infection, larvae were co-injected with 5 μL of dsRNA-CSP3 (1.2 μg/μL) and BmNPV-eGFP (1.0 × 10^6.2^ TCID_50_/mL) to investigate the ELA behavior at 96 hpi. Silkworms injected with dsRNA-DsRed and infected with BmNPV were used as control for dsRNA treatment. Silkworm larvae without dsRNA injections that were infected with BmNPV were used as positive control. Each group contained three to five silkworm larvae, and at least two independent animal experiments were performed. Statistical analyses were performed using Prism 8 software (GraphPad).

### Behavioral assay for enhanced locomotory activity

To quantify ELA behavior, locomotion assays were performed according to the previously described method [17,22]. Briefly, newly molted fifth instar larvae treated as described above were placed in the center of a piece of paper marked with concentric circles (the radius of each circle was 5 cm longer than the previous circle, with a maximum radius of 40 cm in A0 paper). Photographs were taken by a digital camera at 0 s, 30 s, 1 min and 2 min after release. The distance moved during each time interval was determined using ImageJ software and summed up to derive the total distance traveled in 2 min. Statistical analyses were performed using Prism 8 software (GraphPad Prism).

### Sample preparation for lipidomics profiling and neurotransmitter detection

Newly molted fifth-instar silkworm larvaes were injected with either 10 μL of BmNPV-eGFP (1 × 10^6.2^ TCID_50_/mL) or phosphate buffered saline (PBS) (Negative Control, NC). At 96 hours after virus injection, head samples were collected from pools of six BmNPV-infected silkworms that exhibited obvious ELA behavior as one infection sample. The heads of six control silkworms were mixed into one control sample. Three separate samples were each collected for the infection and control groups. Head samples were cleaned with DEPC water, flash frozen and stored in liquid nitrogen until further use.

### Widely targeted lipidomic profiling

Samples (approximately 20 mg) were homogenized under liquid nitrogen and extracted with methyl tert-butyl ether:methanol (3:1, v/v) mixture containing internal standards. After mixing for 15 min, 200 μL of water was added. The samples were vortexed for 1 min and centrifuged at 12,000 rpm for 10 min. Two hundred μL of the upper organic layer was collected and evaporated using a vacuum concentrator. The dry extract was dissolved in 200 μL acetonitrile:isopropylalcohol (1:1, v/v) for LC-MS/MS analysis. An Ultra-Performance Liquid Chromatography Mass Spectrometry (UPLC-MS/MS) system at Wuhan Metware Biotechnology and Banian Medical Technology was used [76]. The raw mass spectrometry data acquisition and processing of all samples was performed using Analyst software 1.6.3 (AB Sciex, Foster City, CA, USA). Characterization of lipid molecular species was carried out with a self-built database, MWDB, including more than 3000 lipid molecules (Metware Biotechnology Co., Ltd., Wuhan, China). Lipid quantification was carried out using multiple reaction monitoring (MRM) with triple quadrupole mass spectrometry. After obtaining the lipid mass spectrometry data of different samples, the mass spectral peaks of the same lipid in different samples were integrated and corrected [77].

Multivariate statistical analyses including Principal Component Analysis (PCA) and Orthogonal partial least squares discriminant analysis (OPLS-DA) were used to distinguish differences in the silkworm head lipidome. For two-group analysis, differential metabolites were determined by Variable Importance of Projection (VIP > 1) and P-value (P-value < 0.05, Student’s t test). The KEGG database [78] was used to annotate lipids and to identify metabolic pathways containing differential lipids. KEGG pathway enrichment analysis was performed, in which the Rich Factor was the ratio of the number of differential lipids in the corresponding pathway to the total number of lipids annotated by the pathway.

Total ion current plots, analysis of different quality control samples and inspection of retention time and peak intensity were performed to ascertain the repeatability and validity of the data. When the coefficient of variation (CV) was plotted against the percentage of peaks, the proportions of substances with CV values less than 0.5 and 0.3 in samples were higher than 85% and 65%, respectively, indicating that the experimental data were very stable (S8A Fig). 2D-PCA plots showed the clear separation of negative control from BmNPV infection data (S8B Fig).

An overview of the different lipid subclasses and their proportions in the head samples of silkworm larvae is presented in S9A Fig, together with a cluster heat map for all samples (S9B Fig).

The lipid group data were analyzed according to the OPLS-DA model, and the scores of each group were plotted to further demonstrate the differences between the groups [79]. S10A and S10B Fig presents the OPLS-DA_scorePlot and the S-Plot between BmNPV vs NC, respectively, together with an OPLS-DA verification diagram (S10C Fig). The Fold Change (FC) value of the lipids in the BmNPV infected group was calculated and the difference in lipid content was arranged from small to large according to the size of the FC value (S10D Fig). S1 Fig provides an overview of all lipids with statistically significant differential abundance, both as Volcano plot (A) and a differential clustering heat map (B).

### Neurotransmitter profiling

Samples (approximately 50 mg) were mixed with 500 µL of 70% methanol/water, vortexed for 3 min at 2500 r/min and subsequently centrifuged at 12000 r/min for 10 min at 4°C. Three hundred μL of supernatant was pipetted into a new centrifuge tube and incubated at -20°C for 30 min. After centrifugation (12000 r/min for 10 min at 4°C), 200 μL of supernatant was used for further LC-MS analysis. Neurotransmitter profiling was performed by Wuhan Metware Biotechnology using UPLC-MS/MS [80]. Briefly, data from all samples (including standards) were collected in MRM (Multiple Reaction Monitoring) mode, and mass spectrometry data were processed using Analyst software 1.6.3 and MultiQuant software 3.0.3 software. Subsequently, the mass spectrometry data of different standards were used to calculate the content of each neurotransmitter in the samples by plotting the standard curves separately. PCA statistical analysis was utilized to distinguish the differences in neurotransmitters in the heads of silkworms. For two-group analysis, differential metabolites were determined by fold change ≤ 0.5 and ≥ 2.

Sample quality control analysis provided evidence of reliable data, as evidenced by the Empirical Cumulative Distribution Function, that plots the CV against the percentage of peaks (S11A Fig). PCA analysis showed distinct segregation of negative control and BmNPV infected samples (S11B Fig).

### Modeling of BmCSP3 structure

To obtain a homology model of BmCSP3 we searched for its sequence (UniProt ID: Q3LBA2, residues 19–127 lacking the signal peptide 1–18) using the SWISS-MODEL server [42]. The top-ranked CSPs with known structure displayed sequence identity 52–54% (S4 File). The structure of CSPA6 from *Mamestra brassicae* (CSPMbraA6; also called CSP2) [44] was selected as template for homology modeling of BmCSP3 using MODELLER v10.4 [81]. We prepared a ligand-free model of BmCSP3 based on PDB ID: 1kx9 [44] and a lipid-bound model based on PDB ID: 1n8v [35], considering the potential that BmCSP3 can undergo large conformational changes similar to CSPMbraA6. The top-ranked model according to DOPE score was used either without any ligands, or including the 3 molecules of 12-bromo-1-dodecanol that were resolved in the X-ray structure of CSPMbraA6 [35] after superposition with the template structure (PDB ID: 1n8v).

### Molecular dynamics (MD) simulations of BmCSP3 models

To investigate the stability of the obtained BmCSP3 models we employed atomistic molecular dynamics simulations (MDs) in explicit solvent, without any restraints. We studied both the ligand-free and lipid-bound models of BmCSP3 in addition to the corresponding crystallographic structures of CSPMbraA6, all under the same computational parameters for comparison. MD calculations were carried out using the GPU-accelerated version of PMEMD in AMBER v22 [82]. Briefly, the simulation systems were prepared using the LEaP module of AMBER and the latest protein force field ff14SB [83]. The ligands were prepared using the ANTECHAMBER module of AMBER, with AM1-BCC charges and the General Amber Force Field [84]. Each protein was placed in a truncated octahedral box comprising TIP3P waters and having a buffer distance of 10 Å around the solute. Then Na^+^ and Cl^–^ ions were added to neutralize the total charge and obtain an ion concentration of 0.15 M. Energy minimization was performed in 3 steps, first by applying positional restraints of 10 Kcal × mol^−1^ × Å^−2^ to all heavy atoms, then only to C_α_ atoms and lastly without any restraints. Equilibration of the solvent molecules comprised of heating the system from 100 to 300 K within 100 ps under constant volume (NVT ensemble) and then simulation for 400 ps at 300 K under a constant pressure of 1 bar (NPT ensemble), with positional restraints of 100 Kcal × mol^−1^ × Å^−2^ to all solute atoms. Following an energy minimization step, equilibration of the density at 300 K was carried out under constant pressure of 1 bar through 10 ns of simulation, in which the positional restraints were gradually removed within the first 1 ns. Two independent production simulations for each ligand-free and ligand-bound forms of BmCSP3 and CSPMbraA6 were then performed in the NPT ensemble for 1 μs at 2 fs time-step. The particle mesh Ewald summation method for treatment of long-range electrostatic interactions was used with the real space truncated at 9 Å, in combination with the SHAKE algorithm to constrain hydrogen atoms at their equilibrium distance. The Berendsen weak-coupling algorithm with a relaxation time of 1 ps was used to regulate pressure, and the Langevin thermostat with a collision frequency of 1 or 2 ps^–1^ (for run-1 and run-2, respectively) was used to regulate the temperature of the systems. MD trajectories were collected every 10 ps from a total of 1.01 μs, including the equilibration stage, resulting in a total of 101,000 frames for each system. Trajectories were analyzed using the CPPTRAJ v17 module of AMBER [85] and figures were generated using the open-source version 2.5 of PyMOL.

### Docking of neurotransmitters and selected lipids into the BmCSP3 models

The chemical structures of 9 neurotransmitters employed for profiling and of a selection of 15 lipids from the lipidomics profiling were retrieved in SMILES representations from PubChem [86]. A 3D conformer of each ligand was generated using OMEGA v4.2 [87], which was then prepared for docking using AutoDock Tools v1.5.7 [88]. The models of BmCSP3 in the closed (ligand-free) and open (ligand-bound) forms were prepared for docking using AutoDockTools and a search space of 30 × 30 × 30 (Å) that included all protein molecules. Docking calculations were carried out using AutoDock Vina v1.2.3 [89] with default parameters, except for the exhaustiveness level that was set to 20.

### Chemicals used in binding assays

Compounds tested in this study are listed in Table 1. N-Acetylserotonin, Serotonin hydrochloride, L-Glutamine, L-Kynurenine, L-Lysine and DL-Ornithine hydrochloride were purchased from Fluorochem; Tryptamine from Alfa Aesar; and Homogentistic Acid from Tokyo Chemical Industry (TCI). Glycine was obtained from the inventory of the Institute of Chemical Biology, National Hellenic Research Foundation (NHRF). 9(S)-HOTrE (CAS: 89886-42-0) was purchased from Cayman chemical and (±)9(10)-EpOME (CAS: 16833-56-0) from MedChemExpress.

### BmCSP3 protein expression and purification

BmCSP3 (residues 19–109 of UniProt entry Q3LBA2) was expressed in *E. coli* [BL21(DE3)] cells (Thermo Fisher Scientific) transformed with a pET22-BmCSP3 expression vector. BmCSP3 was purified from clarified cell lysate using a 6 mL Resource-Q column equilibrated with 50 mM Tris-HCl, pH 8.5. The protein was eluted with a NaCl gradient (20–1000 mM) in the same buffer, concentrated, and subsequently applied to a Superdex 75 16/60 column (GE Healthcare) equilibrated with 10 mM Tris-HCl, pH 8.0, and 200 mM NaCl. The resulting protein was delipidated by the addition of 200 μL LIPIDEX 1000 (PerkinElmer) on a 0.22 μm spin filter and concentrated to 15–20 mg/mL for use in the fluorescence experiments.

### Fluorescence competitive binding assays

The dissociation constant (Kd) of the fluorescent probe 1-NPN (N-Phenyl-1-naphthylamine) was determined in solutions containing 4 μΜ BmCSP3 in 20 mM Tris-HCl pH 8.0, 100 mM NaCl by addition of 0 το 20 μM 1-NPN. To determine the ligands’ Ki, 1-NPN displacement was measured in solutions containing 4 μΜ BmCSP3, 10 μΜ 1-NPN and ligand concentrations ranged from 0-20 μΜ and 0–32 μΜ, for the tested neurotranmiters and lipids, respectively. Control samples containing the ligand and 1-NPN, but lacking protein, were run in parallel to determine background fluorescence, which was subtracted from the experimental data. All experiments were performed in technical triplicate.

Measurements were taken using a Varioskan Flash fluorimeter plate reader (Thermo Scientific) or a BioTek Synergy H1 plate reader (Agilent) at 25^°^C, in black 96-well plates (Greiner, Bio-One), with a final assay volume of 300μL. Fresh aliquots of the ligands under study were prepared and dissolved in DMSO. The final concentration of the organic solvent did not exceed 2.8% for neurotransmitters and 4.6% for lipids. The probe was excited at 337 nm and emission spectra were recorded between 360 and 500 nm.

The determination of dissociation constants and the visualization of the binding curves was carried out using the non-linear regression analysis program GraphPad Prism 8 (www.graphpad.com). The Kd of 1-NPN for BmCSP3 was determined to be 5.8 μΜ by fitting the fluorescence data to a single-binding site saturation model described by the equation


Y=Bmax*X/(Kd+X),\]

where Bmax is the maximum specific bindings to the site, X is the total ligand concentration in the assay and Kd is the equilibrium dissociation constant. The affinities of the ligands were assessed by titration of solutions containing constant concentrations of CSP3 (4 μΜ) and 1-NPN (10 μΜ). The fluorescence decrease data were fitted to the “One site-Fit logEC50” model under competitive binding conditions, using the equation listed below


Y=Bottom + (Top-Bottom)/(1+10 ^ (X-LogEC50))\]

where, Top and Bottom are plateaus and logEC50 is the log of the ligand that results in %Fluorescence intensity half-way between the Bottom and Top value.

Given that the bottom values never reaches zero and varied among the different experiments, the ligand concentration that reduced the initial fluorescence by 50% (EC50int) was estimated from the displacement binding curves by interpolation [90], using the built-in interpolation function of GraphPad Prism. Subsequently, the ligand’s dissociation constant *(Ki)* was determined using the Cheng-Prusoff equation:


$$Ki,ligand=\frac{EC\text{5}\text{0}int}{\text{1}+\frac{\left[\text{1}NPN\right]}{Kd,\text{1}NPN}}$$

where, [1-NPN] is the concentration of the fluorescent probe (10 μM), and *K*d,1 NPN is its dissociation constant (5.8 μM).

## Supporting information

S1 Fig Identification of lipids with differential abundance between uninfected and BmNPV-infected head samples. (A) Differential lipid volcano plot. Each dot represents a lipid and the size of the dots represents the Variable Importance in Projection (VIP) value. Lipids with statistically significant increase or decrease in abundance are colored in red or green, respectively. (B) Differential lipid clustering heat map. Differential values after normalization (Z-scores) are presented as color intensity (red represents increased abundance, green represents decreased abundance). The different classes of lipids are indicated (GP, glycerophospholipids, GL, glycerolipids; FA, fatty acids; SP, sphingolipids; ST, sterols). (C) Differential lipid pathway classification diagram. In the ordinate, metabolic pathways are displayed while in the abscissa the number and proportion of differential lipids is indicated. (D) Differential lipid pathway enrichment map. The abscissa represents the Rich Factor corresponding to each pathway and the ordinate displays the pathway name (sorted according to P-value). The color and size of the dots reflect the P-value size and the number of enriched differential lipids, respectively.
(TIF)

S2 Fig Superposition of BmCSP3 models in the closed and open conformation (alternative view). Superposition of the BmCSP3 homology models that were based on the ligand-free/closed (cyan) and ligand-bound/open (green) forms of CSPMbraA6, similar to Fig 6C. In this alternative view, ligands are omitted to exhibit the two disulfide bridges (Cys31-Cys38 and Cys57-Cys60), as well as the outward shift of helix α3 that results in a significant opening of the ligand-binding cavity.
(TIF)

S3 Fig Models of CSP3 in two different conformations of the closed (ligand-free) form showing different rotamers of Tyr28. Homology models of BmCSP3 based on the two conformations that were resolved in the X-ray crystal structure of CSPMbraA6 (ligand-free form). The two models are indicated as chains A and B, according to the two crystallographic molecules that were resolved in the asymmetric unit (PDB ID: 1kx9). Chains are superimposed and color-coded to exhibit the two different side-chain rotamers of Tyr28 that gives rise to extension of the hydrophobic ligand-binding cavity (yellow surface).
(TIF)

S4 Fig Interaction model of BmCSP3 with multiple lipid molecules. Binding cavity residues that interact with the three 12-bromo-1-dodecanole molecules in the X-ray structure of CSPMbraA6 (PDB ID: 1n8v) and the corresponding residues of the BmCSP3 homology model. Inset on top is their sequence alignment with the six helices designated α1–α6. Similar residues are labeled according to the numbering of CSPMbraA6 (gray C atoms), whereas different residues are also indicated by the corresponding residue of BmCSP3 (green C atoms). Ligands (BD1–BD3) are color-coded with yellow C atoms, whereas all the O and N atoms are colored red and blue, respectively. The main interactions of the three ligands with BmCSP3 are the following; BD1: H-bond with D11, hydrophobic interactions with V13, L15, V18, L25, I29 and I32; BD2: H-bond with Y100, hydrophobic interactions with L45, I49, A52, L53 and Y6; BD3: H-bond with D91, hydrophobic interactions with L15, L45, L53, V71, I72 and W83.
(TIF)

S5 Fig Docking of selected lipids in the binding cavity of the BmCSP3 model. (A) Lowest energy poses of the 15 lipids employed for docking in the closed conformation of BmCSP3 model. Inset is the list of lipids sorted by the VINA docking score (in kcal/mol). (B) Lowest energy poses of the 15 lipids employed for docking in the open state model of BmCSP3. Inset is the list of lipids sorted by the VINA docking score (in kcal/mol).
(TIF)

S6 Fig Docking of neurotransmitter-like molecules in the binding cavity of the BmCSP3 model. (A) Lowest-energy bound poses of the 9 neurotransmitter-related compounds docked in chain B of the closed conformation of BmCSP3 model. (B) Lowest-energy bound poses of the 9 neurotransmitter-related compounds docked in the open conformation of BmCSP3 model. (C) VINA scores of the lowest energy pose obtained from docking of the 9 neurotransmitter-related compounds in the models of BmCSP3 closed states (chain A and B) and open state. The compounds are ranked according to the average scores.
(TIF)

S7 Fig 7*Binding of* (±)9(10)-EpOME *to CSP3.* A reduction in fluorescence (11.9%) was observed upon addition of 0.1 μM of the compound. At higher concentrations, a concentration-dependent increase in 1-NPN fluorescence was observed, suggesting possible micelle formation. Fluorescence measurements were conducted using 4 μM CSP3 and a constant 1-NPN concentration (10 μM), with (±)9(10)-EpOME ranging from 0 to 16 μM.
(TIF)

S8 Fig Quality control data of widely targeted lipidomics samples. (A) Coefficient of variation (CV) distribution chart of each group of silkworm head samples collected at 96 hpi. The abscissa represents the CV value and the ordinate represents the proportion of the total number of substances. Different colors represent different grouped samples (NC, negative control; BmNPV, infected; QC, quality control). (B) PCA score plot of mass spectrometry data of each group of silkworm head samples collected at 96 hpi. PC1 and PC2 represent the first and second principal component, respectively. The percentage represents the explanation rate of the data set by the principal component. Different colors represent different grouped samples (NC, negative control; BmNPV, infected; QC, quality control).
(TIF)

S9 Fig Overview of lipid detection by widely targeted lipidomics. (A) Lipid subclass composition donut diagram. Each color represents a lipid subcategory and the area indicates the proportion of that category. For classification of lipids see [91] and [92]. (B) Cluster analysis of detected lipids. After unit variant scaling, heat map analysis was carried out on all samples. Differential values after normalization (Z-scores) are presented as color intensity (red represents increased abundance, green represents decreased abundance). The different classes of lipids are indicated (GP, glycerophospholipids, GL, glycerolipids; FA, fatty acids; SP, sphingolipids; ST, sterols).
(TIF)

S10 Fig Overview of differential lipid analysis by widely targeted lipidomics. (A) OPLS-DA score plot. The abscissa and ordinate represent orthogonal principal components. Samples of different groups (NC, negative control; BmNPV, infected) are shown indifferent colors. (B) OPLS-DA S-plot. The abscissa and ordinate represent the covariance and the correlation coefficient, respectively, between the principal components and lipids. The closer the lipids are to the upper right corner and lower left corner, the greater the difference. Significantly, red points indicate that the Variable Importance in Projection (VIP) value of these lipids is greater than 1 and green points indicate that the VIP value of these lipids is less than or equal to 1. (C) OPLS-DA verification diagram. The values of R^2^X, R^2^Y (explanation rate of the X and Y matrices, respectively) and Q^2^ (representing the predictive ability of the model) are plotted. The abscissa represents the model R^2^X, R^2^Y and Q^2^ values and the ordinate is the frequency of the model classification effect. Permutation tests are performed to determine the predictive capability of the OPLS-DA model. Excellent values (close to 1) are obtained for R^2^Y and Q^2^ together with highly significant P value. (D) Dynamic distribution chart of lipid content differences. The cumulative number of substances arranged in ascending order is plotted against the logarithmic value of the fold difference. The green and red points represent the top 10 substances that were downregulated and up-regulated, respectively.
(TIF)

S11 Fig Quality control data of targeted neurotransmitter metabolomics samples. (A) Coefficient of variation (CV) distribution chart of each group of samples. The abscissa represents the CV value and the ordinate represents the proportion of the total number of substances. Different colors represent different grouped samples (NC, negative control; BmNPV, infected; QC, quality control). (B) PCA score plot of mass spectrometry data of each group of samples. PC1 and PC2 represent the first and second principal component, respectively. The percentage represents the explanation rate of the data set by the principal component. Different colors represent different grouped samples (NC, negative control; BmNPV, infected; QC, quality control).
(TIF)

S1 File Properties of DEGs between the BmNPV-infected and control groups in each brain cluster at 96 hpi. (XLS)

S2 File Properties of differential lipids that have increased and decreased abundance in the BmNPV-infected group of head samples at 96 hpi. (XLSX)

S3 File Properties of neurotransmitters that have differential abundance between control and BmNPV-infected groups of head samples at 96 hpi. (XLSX)

S4 File Best structural templates of BmCSP3 based on their sequence identity and coverage. (DOCX)

S5 File Primers for dsRNA synthesis, RT-PCR and qPCR detection. (XLSX)

## References

[1] Reichert H, Boyan G. Building a brain: developmental insights in insects. Trends Neurosci. 1997;20(6):258–64. doi: 10.1016/s0166-2236(96)01034-x 9185307

[2] Wang G-B, Zheng Q, Shen Y-W, Wu X-F. Shotgun proteomic analysis of Bombyx mori brain: emphasis on regulation of behavior and development of the nervous system. Insect Sci. 2016;23(1):15–27. doi: 10.1111/1744-7917.12195 25504592

[3] Gan L, Liu X, Xiang Z, He N. Microarray-based gene expression profiles of silkworm brains. BMC Neurosci. 2011;12:8. doi: 10.1186/1471-2202-12-8 21247463 PMC3032748

[4] Han X, Wang R, Zhou Y, Fei L, Sun H, Lai S, et al. Mapping the Mouse Cell Atlas by Microwell-Seq. Cell. 2018;173(5):1307. doi: 10.1016/j.cell.2018.05.012 29775597

[5] Raj B, Wagner DE, McKenna A, Pandey S, Klein AM, Shendure J, et al. Simultaneous single-cell profiling of lineages and cell types in the vertebrate brain. Nat Biotechnol. 2018;36(5):442–50. doi: 10.1038/nbt.4103 29608178 PMC5938111

[6] Wang Q, Peng C, Yang M, Huang F, Duan X, Wang S, et al. Single-cell RNA-seq landscape midbrain cell responses to red spotted grouper nervous necrosis virus infection. PLoS Pathog. 2021;17(6):e1009665. doi: 10.1371/journal.ppat.1009665 34185811 PMC8241073

[7] Croset V, Treiber CD, Waddell S. Cellular diversity in the Drosophila midbrain revealed by single-cell transcriptomics. Elife. 2018;7:e34550. doi: 10.7554/eLife.34550 29671739 PMC5927767

[8] Davie K, Janssens J, Koldere D, De Waegeneer M, Pech U, Kreft Ł, et al. A single-cell transcriptome atlas of the aging drosophila brain. Cell. 2018;174(4):982-998.e20. doi: 10.1016/j.cell.2018.05.057 29909982 PMC6086935

[9] Li H, Janssens J, De Waegeneer M, Kolluru SS, Davie K, Gardeux V, et al. Fly cell atlas: a single-nucleus transcriptomic atlas of the adult fruit fly. Science. 2022;375(6584):eabk2432. doi: 10.1126/science.abk2432 35239393 PMC8944923

[10] Cui Y, Behura SK, Franz AWE. Cellular diversity and gene expression profiles in the male and female brain of Aedes aegypti. BMC Genomics. 2022;23(1):119. doi: 10.1186/s12864-022-08327-9 35144549 PMC8832747

[11] Feng M, Fei S, Zou J, Xia J, Lai W, Huang Y, et al. Single-nucleus sequencing of silkworm larval brain reveals the key role of lysozyme in the antiviral immune response in brain hemocytes. J Innate Immun. 2024;16(1):173–87. doi: 10.1159/000537815 38387449 PMC10965234

[12] Hughes DP, Libersat F. Neuroparasitology of parasite-insect associations. Annu Rev Entomol. 2018;63:471–87. doi: 10.1146/annurev-ento-020117-043234 29324045

[13] Goulson D. Wipfelkrankheit: modification of host behaviour during baculoviral infection. Oecologia. 1997;109(2):219–28. doi: 10.1007/s004420050076 28307172

[14] van Houte S, Ros VID, Mastenbroek TG, Vendrig NJ, Hoover K, Spitzen J, et al. Protein tyrosine phosphatase-induced hyperactivity is a conserved strategy of a subset of baculoviruses to manipulate lepidopteran host behavior. PLoS One. 2012;7(10):e46933. doi: 10.1371/journal.pone.0046933 23077534 PMC3471939

[15] Kamita SG, Nagasaka K, Chua JW, Shimada T, Mita K, Kobayashi M, et al. A baculovirus-encoded protein tyrosine phosphatase gene induces enhanced locomotory activity in a lepidopteran host. Proc Natl Acad Sci U S A. 2005;102(7):2584–9. doi: 10.1073/pnas.0409457102 15699333 PMC548987

[16] Gasque SN, van Oers MM, Ros VI. Where the baculoviruses lead, the caterpillars follow: baculovirus-induced alterations in caterpillar behaviour. Curr Opin Insect Sci. 2019;33:30–6. doi: 10.1016/j.cois.2019.02.008 31358192

[17] Kokusho R, Katsuma S. Bombyx mori nucleopolyhedrovirus ptp and egt genes are dispensable for triggering enhanced locomotory activity and climbing behavior in Bombyx mandarina larvae. J Invertebr Pathol. 2021;183:107604. doi: 10.1016/j.jip.2021.107604 33971220

[18] Hikida H, Katsuma S. High-resolution analysis of baculovirus-induced host manipulation in the domestic silkworm, Bombyx mori. Parasitology. 2021;148(1):105–9. doi: 10.1017/S0031182020001924 33054893 PMC7808863

[19] Herbison R, Lagrue C, Poulin R. The missing link in parasite manipulation of host behaviour. Parasit Vectors. 2018;11(1):222. doi: 10.1186/s13071-018-2805-9 29615121 PMC5881176

[20] Hughes DP, Libersat F. Parasite manipulation of host behavior. Curr Biol. 2019;29(2):R45–7. doi: 10.1016/j.cub.2018.12.001 30668944

[21] Wang G, Zhang J, Shen Y, Zheng Q, Feng M, Xiang X, et al. Transcriptome analysis of the brain of the silkworm Bombyx mori infected with Bombyx mori nucleopolyhedrovirus: a new insight into the molecular mechanism of enhanced locomotor activity induced by viral infection. J Invertebr Pathol. 2015;128:37–43. doi: 10.1016/j.jip.2015.04.001 25912089

[22] Li Y, Zhang J, Zhao S, Wu X. BmNPV-induced hormone metabolic disorder in silkworm leads to enhanced locomotory behavior. Dev Comp Immunol. 2021;121:104036. doi: 10.1016/j.dci.2021.104036 33545211

[23] Wang G, Na S, Qin L. Screening of Bombyx mori brain proteins interacting with protein tyrosine phosphatase of BmNPV. Arch Insect Biochem Physiol. 2020;105(2):e21732. doi: 10.1002/arch.21732 32783274

[24] Katsuma S, Koyano Y, Kang W, Kokusho R, Kamita SG, Shimada T. The baculovirus uses a captured host phosphatase to induce enhanced locomotory activity in host caterpillars. PLoS Pathog. 2012;8(4):e1002644. doi: 10.1371/journal.ppat.1002644 22496662 PMC3320614

[25] Katsuma S. Phosphatase activity of Bombyx mori nucleopolyhedrovirus PTP is dispensable for enhanced locomotory activity in B. mori larvae. J Invertebr Pathol. 2015;132:228–32. doi: 10.1016/j.jip.2015.11.002 26550695

[26] Effantin G, Kandiah E, Pelosse M. Structure of AcMNPV nucleocapsid reveals DNA portal organization and packaging apparatus of circular dsDNA baculovirus. Nat Commun. 2025;16(1):4844. doi: 10.1038/s41467-025-60152-2 40413174 PMC12103608

[27] van Houte S, Ros VID, van Oers MM. Hyperactivity and tree-top disease induced by the baculovirus AcMNPV in Spodoptera exigua larvae are governed by independent mechanisms. Naturwissenschaften. 2014;101(4):347–50. doi: 10.1007/s00114-014-1160-8 24563099

[28] Hoover K, Grove M, Gardner M, Hughes DP, McNeil J, Slavicek J. A gene for an extended phenotype. Science. 2011;333(6048):1401. doi: 10.1126/science.1209199 21903803

[29] Ros VID, van Houte S, Hemerik L, van Oers MM. Baculovirus-induced tree-top disease: how extended is the role of egt as a gene for the extended phenotype?. Mol Ecol. 2015;24(1):249–58. doi: 10.1111/mec.13019 25443568

[30] Han Y, van Houte S, van Oers MM, Ros VID. Timely trigger of caterpillar zombie behaviour: temporal requirements for light in baculovirus-induced tree-top disease. Parasitology. 2018;145(6):822–7. doi: 10.1017/S0031182017001822 29144213

[31] Li C-H, Poulin R. Alteration of host gene and protein expression by manipulative parasites. Trends Parasitol. 2025;41(2):83–6. doi: 10.1016/j.pt.2024.11.014 39721905

[32] Lizana P, Mutis A, Quiroz A, Venthur H. Insights into chemosensory proteins from non-model insects: advances and perspectives in the context of pest management. Front Physiol. 2022;13:924750. doi: 10.3389/fphys.2022.924750 36072856 PMC9441497

[33] Satija R, Farrell JA, Gennert D, Schier AF, Regev A. Spatial reconstruction of single-cell gene expression data. Nat Biotechnol. 2015;33(5):495–502. doi: 10.1038/nbt.3192 25867923 PMC4430369

[34] Pelosi P, Iovinella I, Zhu J, Wang G, Dani FR. Beyond chemoreception: diverse tasks of soluble olfactory proteins in insects. Biol Rev Camb Philos Soc. 2018;93(1):184–200. doi: 10.1111/brv.12339 28480618

[35] Campanacci V, Lartigue A, Hällberg BM, Jones TA, Giudici-Orticoni M-T, Tegoni M, et al. Moth chemosensory protein exhibits drastic conformational changes and cooperativity on ligand binding. Proc Natl Acad Sci U S A. 2003;100(9):5069–74. doi: 10.1073/pnas.0836654100 12697900 PMC154299

[36] Mutlu AS, Duffy J, Wang MC. Lipid metabolism and lipid signals in aging and longevity. Dev Cell. 2021;56(10):1394–407. doi: 10.1016/j.devcel.2021.03.034 33891896 PMC8173711

[37] Tan ST, Ramesh T, Toh XR, Nguyen LN. Emerging roles of lysophospholipids in health and disease. Prog Lipid Res. 2020;80:101068. doi: 10.1016/j.plipres.2020.101068 33068601

[38] Stith JL, Velazquez FN, Obeid LM. Advances in determining signaling mechanisms of ceramide and role in disease. J Lipid Res. 2019;60(5):913–8. doi: 10.1194/jlr.S092874 30846529 PMC6495170

[39] Longo N, Frigeni M, Pasquali M. Carnitine transport and fatty acid oxidation. Biochim Biophys Acta. 2016;1863(10):2422–35. doi: 10.1016/j.bbamcr.2016.01.023 26828774 PMC4967041

[40] Serotonin, serotonin receptors and their actions in insects. Neurotransmitter. 2015. doi: 10.14800/nt.314

[41] Farooqi MK, Ali M, Amir M. Melatonin and serotonin: their synthesis and effects in insects. Chronobiol Med. 2022;4(1):24–8. doi: 10.33069/cim.2022.0003

[42] Waterhouse A, Bertoni M, Bienert S, Studer G, Tauriello G, Gumienny R, et al. SWISS-MODEL: homology modelling of protein structures and complexes. Nucleic Acids Res. 2018;46(W1):W296–303. doi: 10.1093/nar/gky427 29788355 PMC6030848

[43] Jansen S, Chmelík J, Zídek L, Padrta P, Novák P, Zdráhal Z, et al. Structure of Bombyx mori chemosensory protein 1 in solution. Arch Insect Biochem Physiol. 2007;66(3):135–45. doi: 10.1002/arch.20205 17966128

[44] Lartigue A, Campanacci V, Roussel A, Larsson AM, Jones TA, Tegoni M, et al. X-ray structure and ligand binding study of a moth chemosensory protein. J Biol Chem. 2002;277(35):32094–8. doi: 10.1074/jbc.M204371200 12068017

[45] Llopis-Giménez A, Caballero-Vidal G, Jacquin-Joly E, Crava CM, Herrero S. Baculovirus infection affects caterpillar chemoperception. Insect Biochem Mol Biol. 2021;138:103648. doi: 10.1016/j.ibmb.2021.103648 34536505

[46] He P, Durand N, Dong S-L. Editorial: insect olfactory proteins (From gene identification to functional characterization). Front Physiol. 2019;10:1313. doi: 10.3389/fphys.2019.01313 31681019 PMC6813725

[47] Mosbah A, Campanacci V, Lartigue A, Tegoni M, Cambillau C, Darbon H. Solution structure of a chemosensory protein from the moth Mamestra brassicae. Biochem J. 2003;369(Pt 1):39–44. doi: 10.1042/BJ20021217 12217077 PMC1223053

[48] Campanacci V, Krieger J, Bette S, Sturgis JN, Lartigue A, Cambillau C, et al. Revisiting the specificity of Mamestra brassicae and Antheraea polyphemus pheromone-binding proteins with a fluorescence binding assay. J Biol Chem. 2001;276(23):20078–84. doi: 10.1074/jbc.M100713200 11274212

[49] Jia Q, Zeng H, Zhang J, Gao S, Xiao N, Tang J, et al. The crystal structure of the Spodoptera litura chemosensory protein CSP8. Insects. 2021;12(7):602. doi: 10.3390/insects12070602 34357261 PMC8305471

[50] Tomaselli S, Crescenzi O, Sanfelice D, Ab E, Wechselberger R, Angeli S, et al. Solution structure of a chemosensory protein from the desert locust Schistocerca gregaria. Biochemistry. 2006;45(35):10606–13. doi: 10.1021/bi060998w 16939212

[51] Zhu F, Song D, Chen H, Tang Q, Huo S, Liu X, et al. A lipidome map of the silkworm Bombyx mori: influences of viral infection. J Proteome Res. 2021;20(1):695–703. doi: 10.1021/acs.jproteome.0c00608 33175548

[52] Hu L, Hu Y, Hong A, Guo J, Zhong C, Cai J, et al. Comparison of lipid profiles of male and female silkworm (Bombyx mori) pupae through high-resolution mass spectrometry-based lipidomics and chemometrics. Food Chem. 2024;459:140396. doi: 10.1016/j.foodchem.2024.140396 39024883

[53] Yang F, Xu X, Hu B, Zhang Z, Chen K, Yu Y, et al. Lipid homeostasis is essential for oogenesis and embryogenesis in the silkworm, Bombyx mori. Cell Mol Life Sci. 2024;81(1):127. doi: 10.1007/s00018-024-05173-8 38472536 PMC10933143

[54] Zhu L, Xie Y, Liu C, Cheng J, Shen Z, Liu X, et al. Baculoviruses manipulate host lipid metabolism via adipokinetic hormone signaling to induce climbing behavior. PLoS Pathog. 2025;21(1):e1012932. doi: 10.1371/journal.ppat.1012932 39888969 PMC11819524

[55] Vellosillo T, Martínez M, López MA, Vicente J, Cascón T, Dolan L, et al. Oxylipins produced by the 9-lipoxygenase pathway in Arabidopsis regulate lateral root development and defense responses through a specific signaling cascade. Plant Cell. 2007;19(3):831–46. doi: 10.1105/tpc.106.046052 17369372 PMC1867370

[56] Hayakawa M, Sugiyama S, Takamura T, Yokoo K, Iwata M, Suzuki K, et al. Neutrophils biosynthesize leukotoxin, 9, 10-epoxy-12-octadecenoate. Biochem Biophys Res Commun. 1986;137(1):424–30. doi: 10.1016/0006-291x(86)91227-1 3718512

[57] Zhou W, Song D, Chen H, Tang Q, Yu Q, Huo S, et al. Identification of key metabolic pathways reprogrammed by BmNPV in silkworm Bombyx mori. J Invertebr Pathol. 2022;190:107736. doi: 10.1016/j.jip.2022.107736 35259411

[58] Fei S, Xia J, Huang Y, Zhou M, Xie B, Kong Y, et al. Baculovirus enhances arginine uptake and induces mitochondrial autophagy to promote viral proliferation. PLoS Pathog. 2025;21(7):e1013331. doi: 10.1371/journal.ppat.1013331 40627780 PMC12273963

[59] Leal GM, Leal WS. Binding of a fluorescence reporter and a ligand to an odorant-binding protein of the yellow fever mosquito, Aedes aegypti. F1000Res. 2014;3:305. doi: 10.12688/f1000research.5879.2 25671088 PMC4309172

[60] Sun YF, De Biasio F, Qiao HL, Iovinella I, Yang SX, Ling Y, et al. Two odorant-binding proteins mediate the behavioural response of aphids to the alarm pheromone (E)-ß-farnesene and structural analogues. PLoS One. 2012;7(3):e32759. doi: 10.1371/journal.pone.0032759 22427877 PMC3299684

[61] Zhu J, Ban L, Song L-M, Liu Y, Pelosi P, Wang G. General odorant-binding proteins and sex pheromone guide larvae of Plutella xylostella to better food. Insect Biochem Mol Biol. 2016;72:10–9. doi: 10.1016/j.ibmb.2016.03.005 27001069

[62] Falomir-Lockhart LJ, Cavazzutti GF, Giménez E, Toscani AM. Fatty acid signaling mechanisms in neural cells: fatty acid receptors. Front Cell Neurosci. 2019;13:162. doi: 10.3389/fncel.2019.00162 31105530 PMC6491900

[63] Offermanns S. Free fatty acid (FFA) and hydroxy carboxylic acid (HCA) receptors. Annu Rev Pharmacol Toxicol. 2014;54:407–34. doi: 10.1146/annurev-pharmtox-011613-135945 24160702

[64] Zolezzi JM, Santos MJ, Bastías-Candia S, Pinto C, Godoy JA, Inestrosa NC. PPARs in the central nervous system: roles in neurodegeneration and neuroinflammation. Biol Rev Camb Philos Soc. 2017;92(4):2046–69. doi: 10.1111/brv.12320 28220655

[65] Ziegler AB, Ménagé C, Grégoire S, Garcia T, Ferveur J-F, Bretillon L, et al. Lack of dietary polyunsaturated fatty acids causes synapse dysfunction in the drosophila visual system. PLoS One. 2015;10(8):e0135353. doi: 10.1371/journal.pone.0135353 26308084 PMC4550417

[66] Wallis TP, Venkatesh BG, Narayana VK, Kvaskoff D, Ho A, Sullivan RK, et al. Saturated free fatty acids and association with memory formation. Nat Commun. 2021;12(1):3443. doi: 10.1038/s41467-021-23840-3 34103527 PMC8187648

[67] Jang S, Choi B, Lim C, Lee B, Cho KS. Roles of Drosophila fatty acid-binding protein in development and behavior. Biochem Biophys Res Commun. 2022;599:87–92. doi: 10.1016/j.bbrc.2022.02.040 35176630

[68] Jeffries KA, Dempsey DR, Behari AL, Anderson RL, Merkler DJ. Drosophila melanogaster as a model system to study long-chain fatty acid amide metabolism. FEBS Lett. 2014;588(9):1596–602. doi: 10.1016/j.febslet.2014.02.051 24650760 PMC4023565

[69] Cambiaggi L, Chakravarty A, Noureddine N, Hersberger M. The role of α-Linolenic acid and its oxylipins in human cardiovascular diseases. Int J Mol Sci. 2023;24(7):6110. doi: 10.3390/ijms24076110 37047085 PMC10093787

[70] Farooqui AA, Horrocks LA, Farooqui T. Glycerophospholipids in brain: their metabolism, incorporation into membranes, functions, and involvement in neurological disorders. Chem Phys Lipids. 2000;106(1):1–29. doi: 10.1016/s0009-3084(00)00128-6 10878232

[71] Hishikawa D, Hashidate T, Shimizu T, Shindou H. Diversity and function of membrane glycerophospholipids generated by the remodeling pathway in mammalian cells. J Lipid Res. 2014;55(5):799–807. doi: 10.1194/jlr.R046094 24646950 PMC3995458

[72] Martínez BA, Hoyle RG, Yeudall S, Granade ME, Harris TE, Castle JD, et al. Innate immune signaling in Drosophila shifts anabolic lipid metabolism from triglyceride storage to phospholipid synthesis to support immune function. PLoS Genet. 2020;16(11):e1009192. doi: 10.1371/journal.pgen.1009192 33227003 PMC7721134

[73] Guo W, Wang X, Ma Z, Xue L, Han J, Yu D, et al. CSP and takeout genes modulate the switch between attraction and repulsion during behavioral phase change in the migratory locust. PLoS Genet. 2011;7(2):e1001291. doi: 10.1371/journal.pgen.1001291 21304893 PMC3033386

[74] Yao L, Wang S, Su S, Yao N, He J, Peng L, et al. Construction of a baculovirus-silkworm multigene expression system and its application on producing virus-like particles. PLoS One. 2012;7(3):e32510. doi: 10.1371/journal.pone.0032510 22403668 PMC3293821

[75] Steuerman Y, Cohen M, Peshes-Yaloz N, Valadarsky L, Cohn O, David E, et al. Dissection of influenza infection in vivo by single-cell RNA sequencing. Cell Syst. 2018;6(6):679–91.doi: 10.1016/j.cels.2018.05.008 29886109 PMC7185763

[76] Qin M, Zhu Q, Lai W, Ma Q, Liu C, Chen X, et al. Insights into the prognosis of lipidomic dysregulation for death risk in patients with coronary artery disease. Clin Transl Med. 2020;10(5):e189. doi: 10.1002/ctm2.189 32997403 PMC7522592

[77] Fraga CG, Clowers BH, Moore RJ, Zink EM. Signature-discovery approach for sample matching of a nerve-agent precursor using liquid chromatography-mass spectrometry, XCMS, and chemometrics. Anal Chem. 2010;82(10):4165–73. doi: 10.1021/ac1003568 20405949

[78] Kanehisa M, Goto S. KEGG: kyoto encyclopedia of genes and genomes. Nucleic Acids Res. 2000;28(1):27–30. doi: 10.1093/nar/28.1.27 10592173 PMC102409

[79] Thévenot EA, Roux A, Xu Y, Ezan E, Junot C. Analysis of the human adult urinary metabolome variations with age, body mass index, and gender by implementing a comprehensive workflow for univariate and OPLS statistical analyses. J Proteome Res. 2015;14(8):3322–35. doi: 10.1021/acs.jproteome.5b00354 26088811

[80] Carreño F, Helfer VE, Staudt KJ, Olivo LB, Barreto F, Herrmann AP, et al. Quantification of neurotransmitters in microdialysate samples following quetiapine dosing to schizophrenia phenotyped rats using a validated LC-MS/MS method. J Chromatogr B Analyt Technol Biomed Life Sci. 2020;1155:122282. doi: 10.1016/j.jchromb.2020.122282 32771966

[81] Sali A, Blundell TL. Comparative protein modelling by satisfaction of spatial restraints. J Mol Biol. 1993;234(3):779–815. doi: 10.1006/jmbi.1993.1626 8254673

[82] Salomon-Ferrer R, Götz AW, Poole D, Le Grand S, Walker RC. Routine microsecond molecular dynamics simulations with AMBER on GPUs. 2. explicit solvent particle mesh ewald. J Chem Theory Comput. 2013;9(9):3878–88. doi: 10.1021/ct400314y 26592383

[83] Maier JA, Martinez C, Kasavajhala K, Wickstrom L, Hauser KE, Simmerling C. ff14SB: improving the accuracy of protein side chain and backbone parameters from ff99SB. J Chem Theory Comput. 2015;11(8):3696–713. doi: 10.1021/acs.jctc.5b00255 26574453 PMC4821407

[84] Wang J, Wolf RM, Caldwell JW, Kollman PA, Case DA. Development and testing of a general amber force field. J Comput Chem. 2004;25(9):1157–74. doi: 10.1002/jcc.20035 15116359

[85] Roe DR, Cheatham TE 3rd. PTRAJ and CPPTRAJ: software for processing and analysis of molecular dynamics trajectory data. J Chem Theory Comput. 2013;9(7):3084–95. doi: 10.1021/ct400341p 26583988

[86] Kim S, Chen J, Cheng T, Gindulyte A, He J, He S, et al. PubChem 2023 update. Nucleic Acids Res. 2023;51(D1):D1373–80. doi: 10.1093/nar/gkac956 36305812 PMC9825602

[87] Hawkins PCD, Skillman AG, Warren GL, Ellingson BA, Stahl MT. Conformer generation with OMEGA: algorithm and validation using high quality structures from the protein databank and cambridge structural database. J Chem Inf Model. 2010;50(4):572–84. doi: 10.1021/ci100031x 20235588 PMC2859685

[88] Morris GM, Huey R, Lindstrom W, Sanner MF, Belew RK, Goodsell DS, et al. AutoDock4 and AutoDockTools4: automated docking with selective receptor flexibility. J Comput Chem. 2009;30(16):2785–91. doi: 10.1002/jcc.21256 19399780 PMC2760638

[89] Eberhardt J, Santos-Martins D, Tillack AF, Forli S. AutoDock Vina 1.2.0: new docking methods, expanded force field, and python bindings. J Chem Inf Model. 2021;61(8):3891–8. doi: 10.1021/acs.jcim.1c00203 34278794 PMC10683950

[90] Pelosi P, Zhu J, Knoll W. From radioactive ligands to biosensors: binding methods with olfactory proteins. Appl Microbiol Biotechnol. 2018;102(19):8213–27. doi: 10.1007/s00253-018-9253-5 30054700

[91] Fahy E, Subramaniam S, Brown HA, Glass CK, Merrill AH Jr, Murphy RC, et al. A comprehensive classification system for lipids. J Lipid Res. 2005;46(5):839–61. doi: 10.1194/jlr.E400004-JLR200 15722563

[92] Liebisch G, Fahy E, Aoki J, Dennis EA, Durand T, Ejsing CS, et al. Update on LIPID MAPS classification, nomenclature, and shorthand notation for MS-derived lipid structures. J Lipid Res. 2020;61(12):1539–55. doi: 10.1194/jlr.S120001025 33037133 PMC7707175

