# Supplementary figures and images for: Chemosensory protein 3 is a brain host factor for the induction of enhanced-locomotory activity in the BmNPV-silkworm infection model
Source: PLoS Pathog. 2025 Dec 1;21(12):e1013701. doi: 10.1371/journal.ppat.1013701 (PMC12688159; doi:10.1371/journal.ppat.1013701)

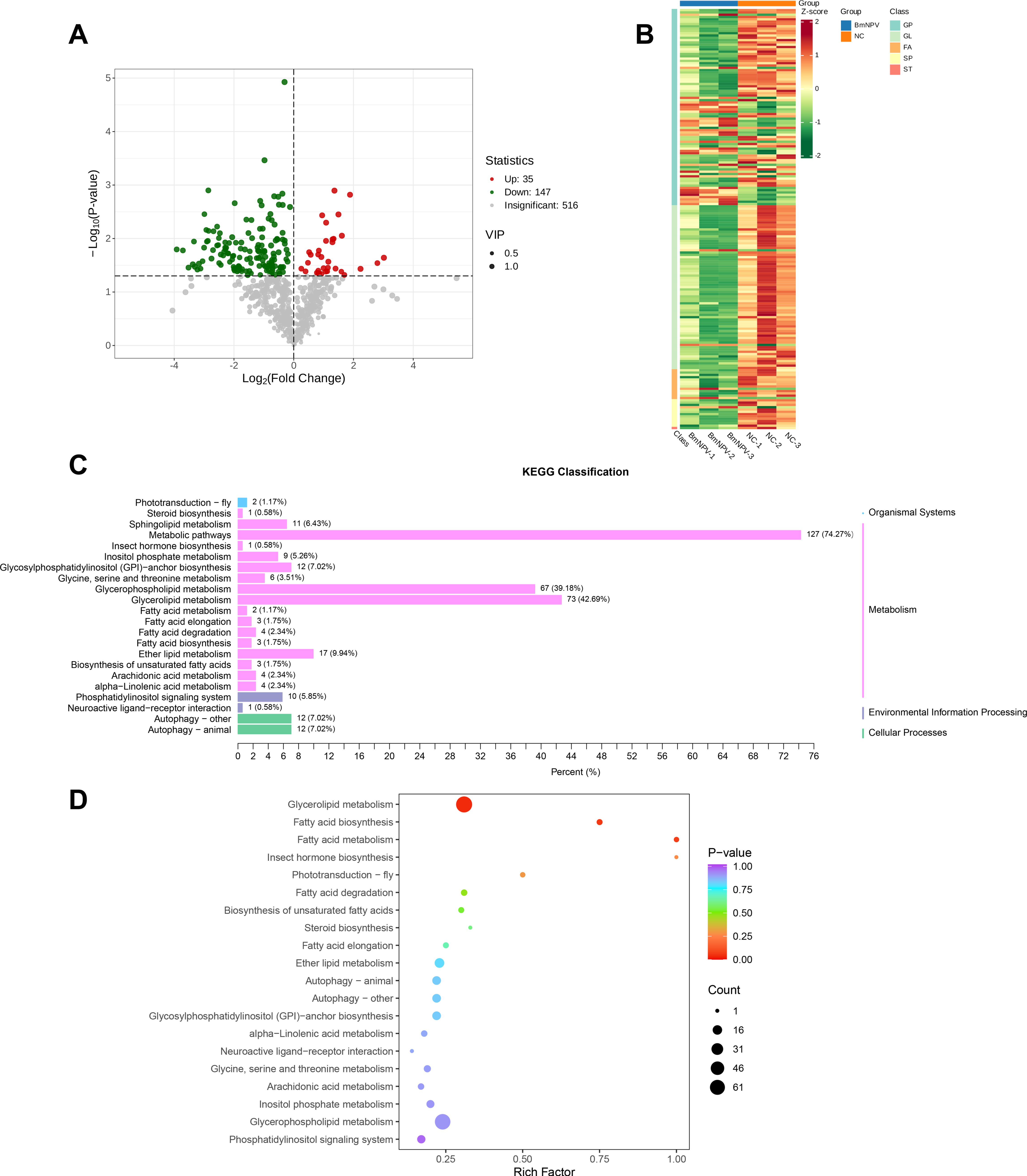

Supplement: S1 Fig — (A) Differential lipid volcano plot. Each dot represents a lipid and the size of the dots represents the Variable Importance in Projection (VIP) value. Lipids with statistically significant increase or decrease in abundance are colored in red or green, respectively. (B) Differential lipid clustering heat map. Differential values after normalization (Z-scores) are presented as color intensity (red represents increased abundance, green represents decreased abundance). The different classes of lipids are indicated (GP, glycerophospholipids, GL, glycerolipids; FA, fatty acids; SP, sphingolipids; ST, sterols). (C) Differential lipid pathway classification diagram. In the ordinate, metabolic pathways are displayed while in the abscissa the number and proportion of differential lipids is indicated. (D) Differential lipid pathway enrichment map. The abscissa represents the Rich Factor corresponding to each pathway and the ordinate displays the pathway name (sorted according to P-value). The color and size of the dots reflect the P-value size and the number of enriched differential lipids, respectively. (TIF) [file ppat.1013701.s001.tif]

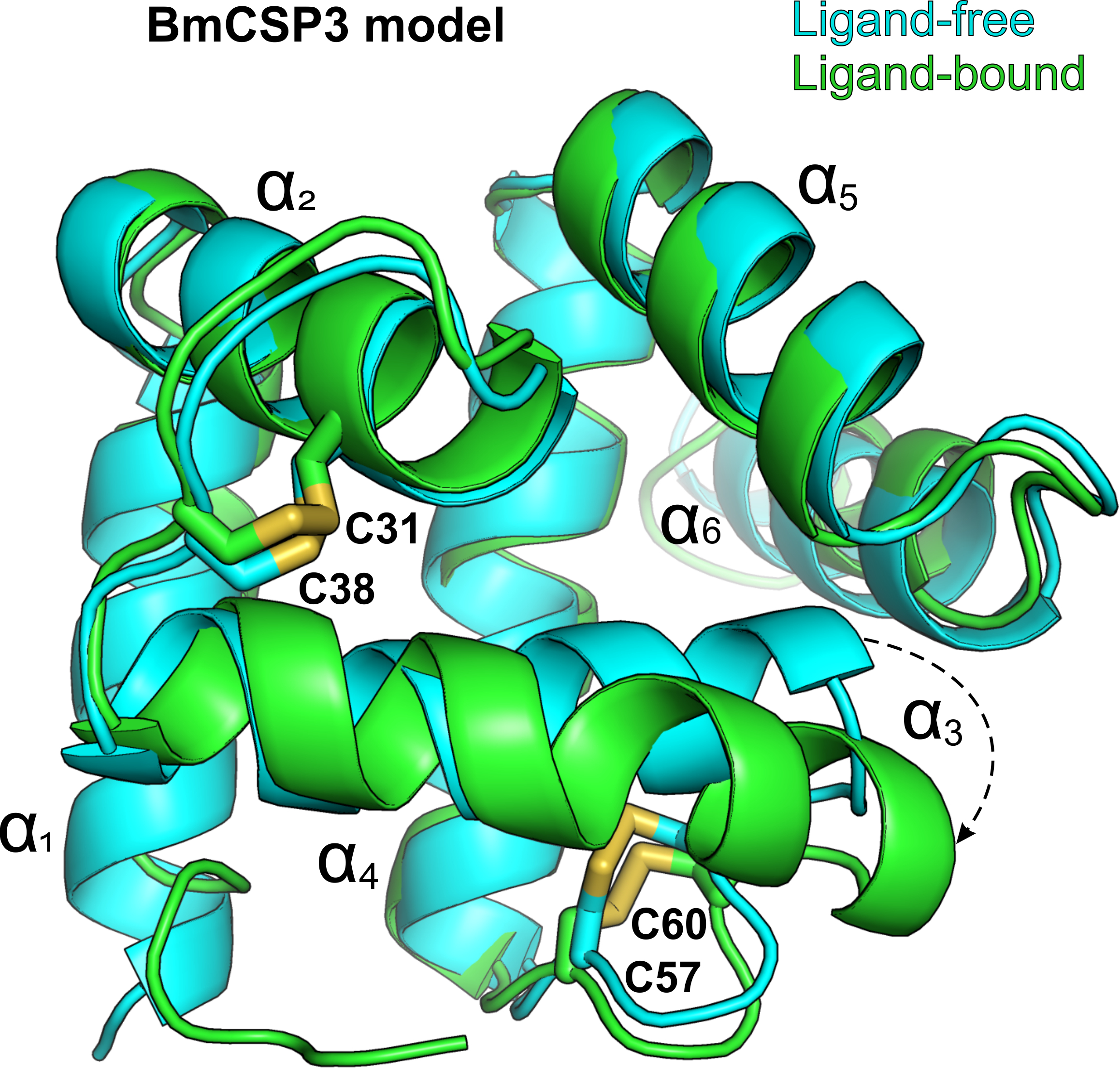

Supplement: S2 Fig — Superposition of the BmCSP3 homology models that were based on the ligand-free/closed (cyan) and ligand-bound/open (green) forms of CSPMbraA6, similar to Fig 6C. In this alternative view, ligands are omitted to exhibit the two disulfide bridges (Cys31-Cys38 and Cys57-Cys60), as well as the outward shift of helix α3 that results in a significant opening of the ligand-binding cavity. (TIF) [file ppat.1013701.s002.tif]

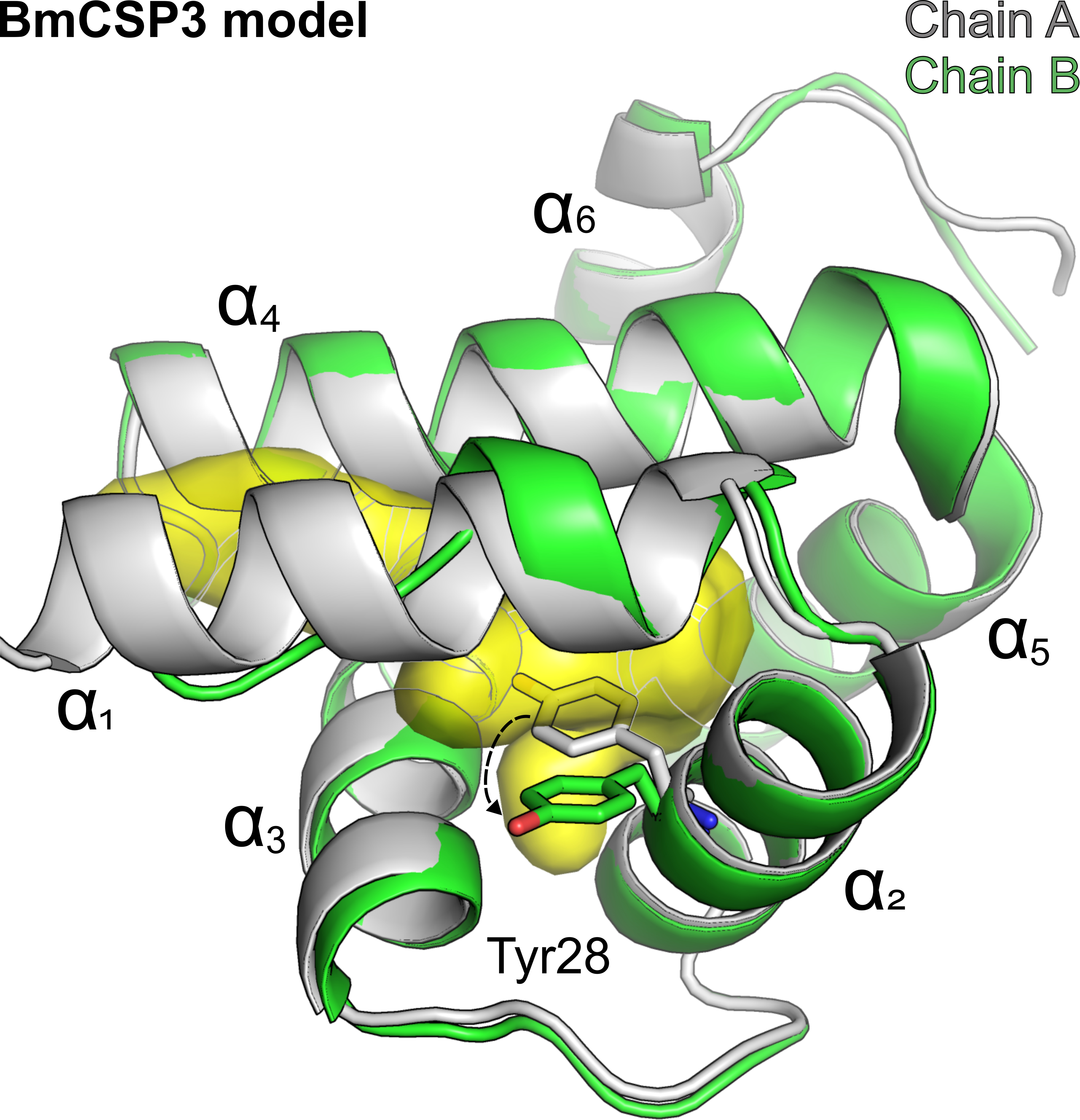

Supplement: S3 Fig — Homology models of BmCSP3 based on the two conformations that were resolved in the X-ray crystal structure of CSPMbraA6 (ligand-free form). The two models are indicated as chains A and B, according to the two crystallographic molecules that were resolved in the asymmetric unit (PDB ID: 1kx9). Chains are superimposed and color-coded to exhibit the two different side-chain rotamers of Tyr28 that gives rise to extension of the hydrophobic ligand-binding cavity (yellow surface). (TIF) [file ppat.1013701.s003.tif]

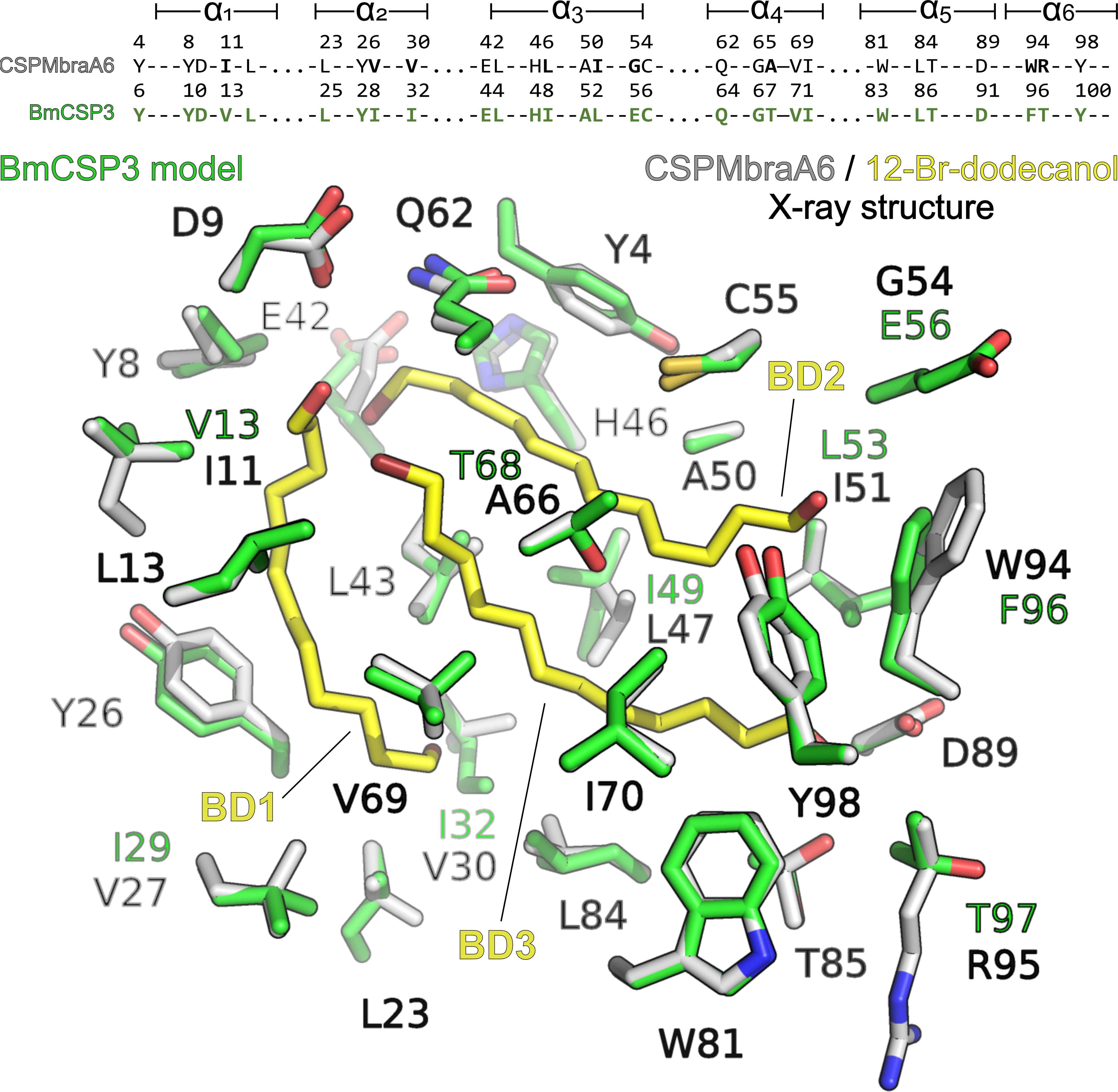

Supplement: S4 Fig — Binding cavity residues that interact with the three 12-bromo-1-dodecanole molecules in the X-ray structure of CSPMbraA6 (PDB ID: 1n8v) and the corresponding residues of the BmCSP3 homology model. Inset on top is their sequence alignment with the six helices designated α1–α6. Similar residues are labeled according to the numbering of CSPMbraA6 (gray C atoms), whereas different residues are also indicated by the corresponding residue of BmCSP3 (green C atoms). Ligands (BD1–BD3) are color-coded with yellow C atoms, whereas all the O and N atoms are colored red and blue, respectively. The main interactions of the three ligands with BmCSP3 are the following; BD1: H-bond with D11, hydrophobic interactions with V13, L15, V18, L25, I29 and I32; BD2: H-bond with Y100, hydrophobic interactions with L45, I49, A52, L53 and Y6; BD3: H-bond with D91, hydrophobic interactions with L15, L45, L53, V71, I72 and W83. (TIF) [file ppat.1013701.s004.tif]

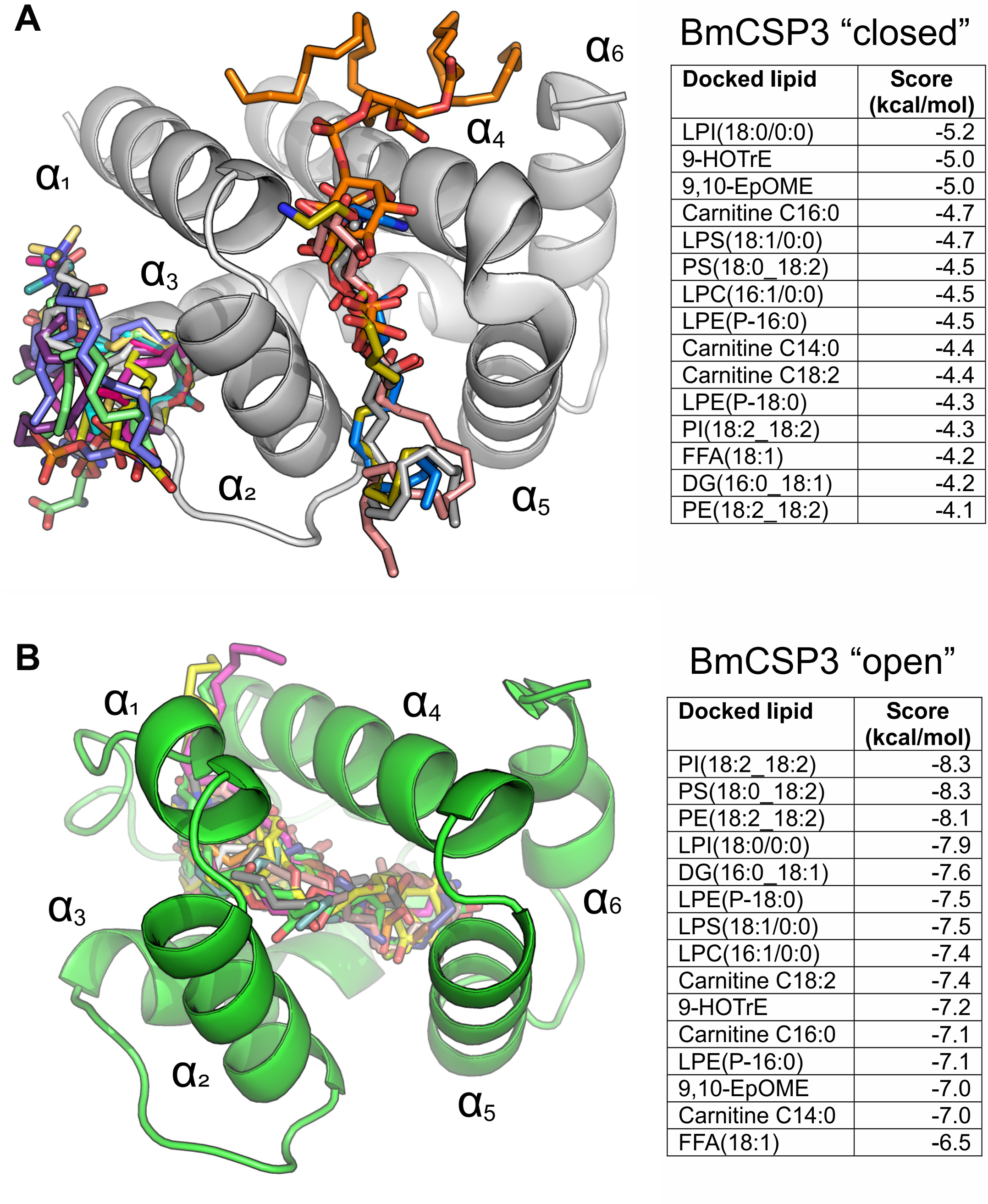

Supplement: S5 Fig — (A) Lowest energy poses of the 15 lipids employed for docking in the closed conformation of BmCSP3 model. Inset is the list of lipids sorted by the VINA docking score (in kcal/mol). (B) Lowest energy poses of the 15 lipids employed for docking in the open state model of BmCSP3. Inset is the list of lipids sorted by the VINA docking score (in kcal/mol). (TIF) [file ppat.1013701.s005.tif]

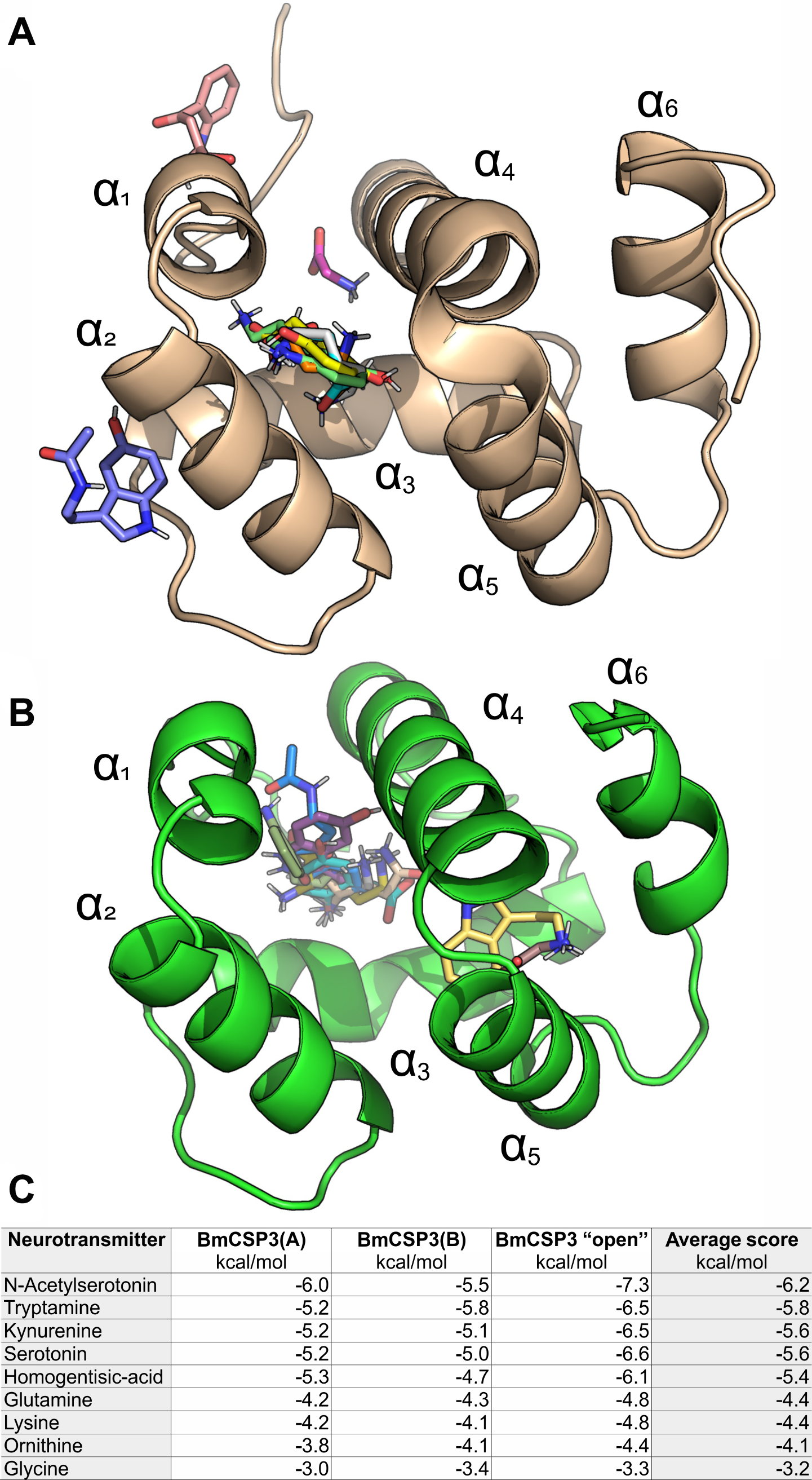

Supplement: S6 Fig — (A) Lowest-energy bound poses of the 9 neurotransmitter-related compounds docked in chain B of the closed conformation of BmCSP3 model. (B) Lowest-energy bound poses of the 9 neurotransmitter-related compounds docked in the open conformation of BmCSP3 model. (C) VINA scores of the lowest energy pose obtained from docking of the 9 neurotransmitter-related compounds in the models of BmCSP3 closed states (chain A and B) and open state. The compounds are ranked according to the average scores. (TIF) [file ppat.1013701.s006.tif]

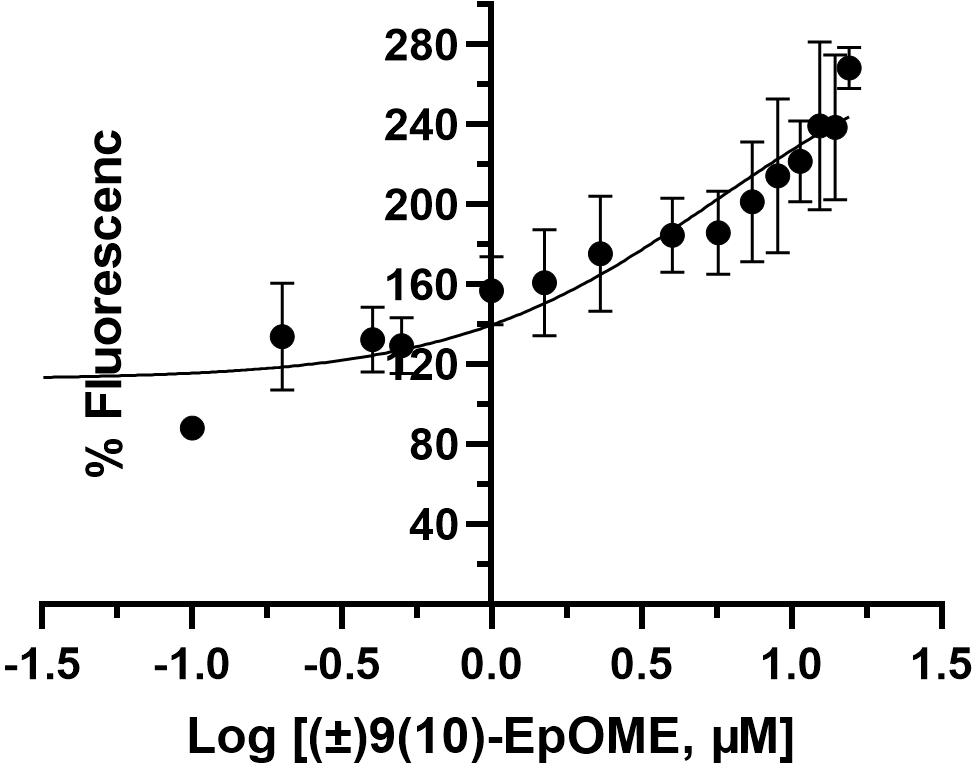

Supplement: S7 Fig — A reduction in fluorescence (11.9%) was observed upon addition of 0.1 μM of the compound. At higher concentrations, a concentration-dependent increase in 1-NPN fluorescence was observed, suggesting possible micelle formation. Fluorescence measurements were conducted using 4 μM CSP3 and a constant 1-NPN concentration (10 μM), with (±)9(10)-EpOME ranging from 0 to 16 μM. (TIF) [file ppat.1013701.s007.tif]

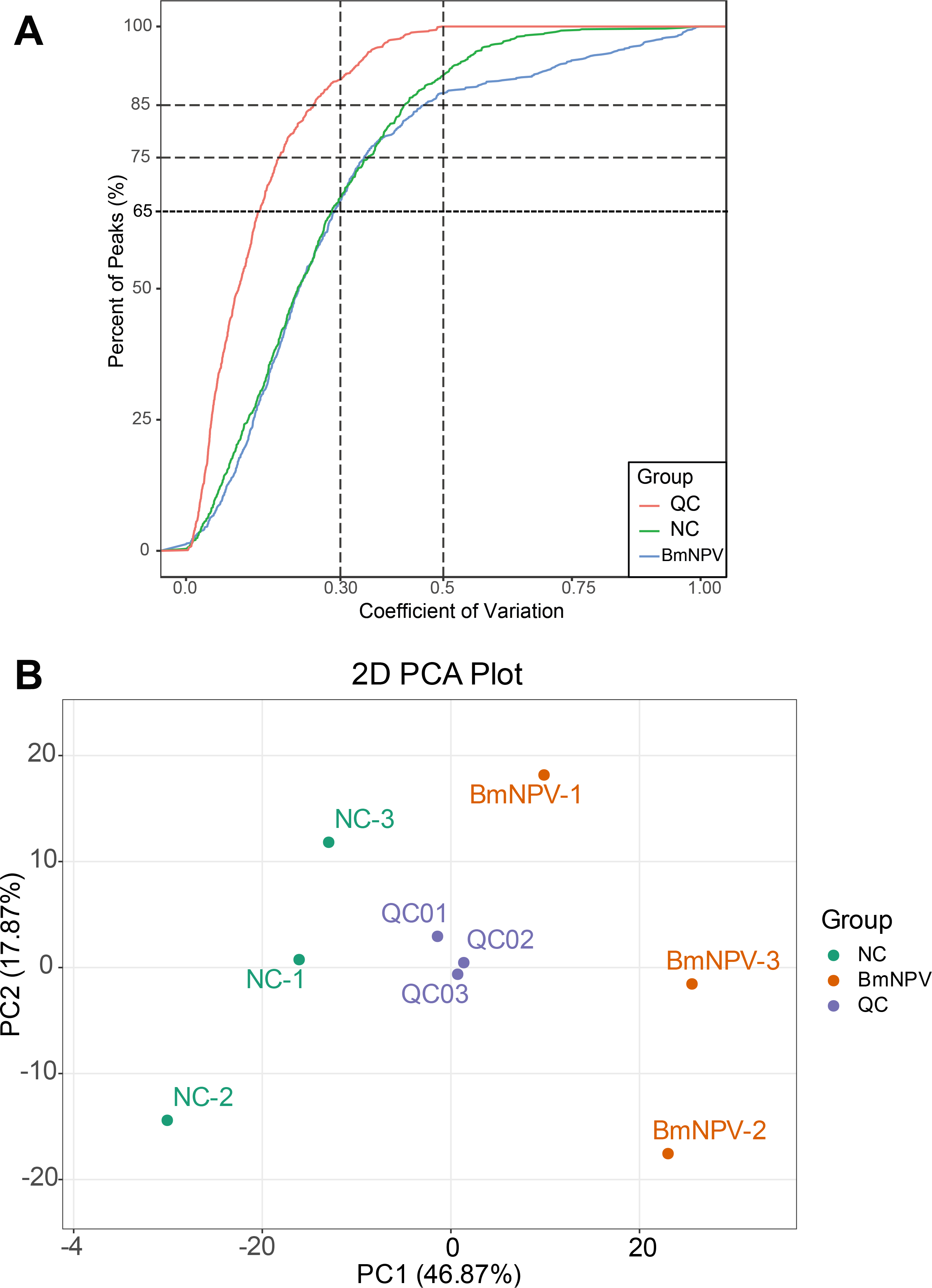

Supplement: S8 Fig — (A) Coefficient of variation (CV) distribution chart of each group of silkworm head samples collected at 96 hpi. The abscissa represents the CV value and the ordinate represents the proportion of the total number of substances. Different colors represent different grouped samples (NC, negative control; BmNPV, infected; QC, quality control). (B) PCA score plot of mass spectrometry data of each group of silkworm head samples collected at 96 hpi. PC1 and PC2 represent the first and second principal component, respectively. The percentage represents the explanation rate of the data set by the principal component. Different colors represent different grouped samples (NC, negative control; BmNPV, infected; QC, quality control). (TIF) [file ppat.1013701.s008.tif]

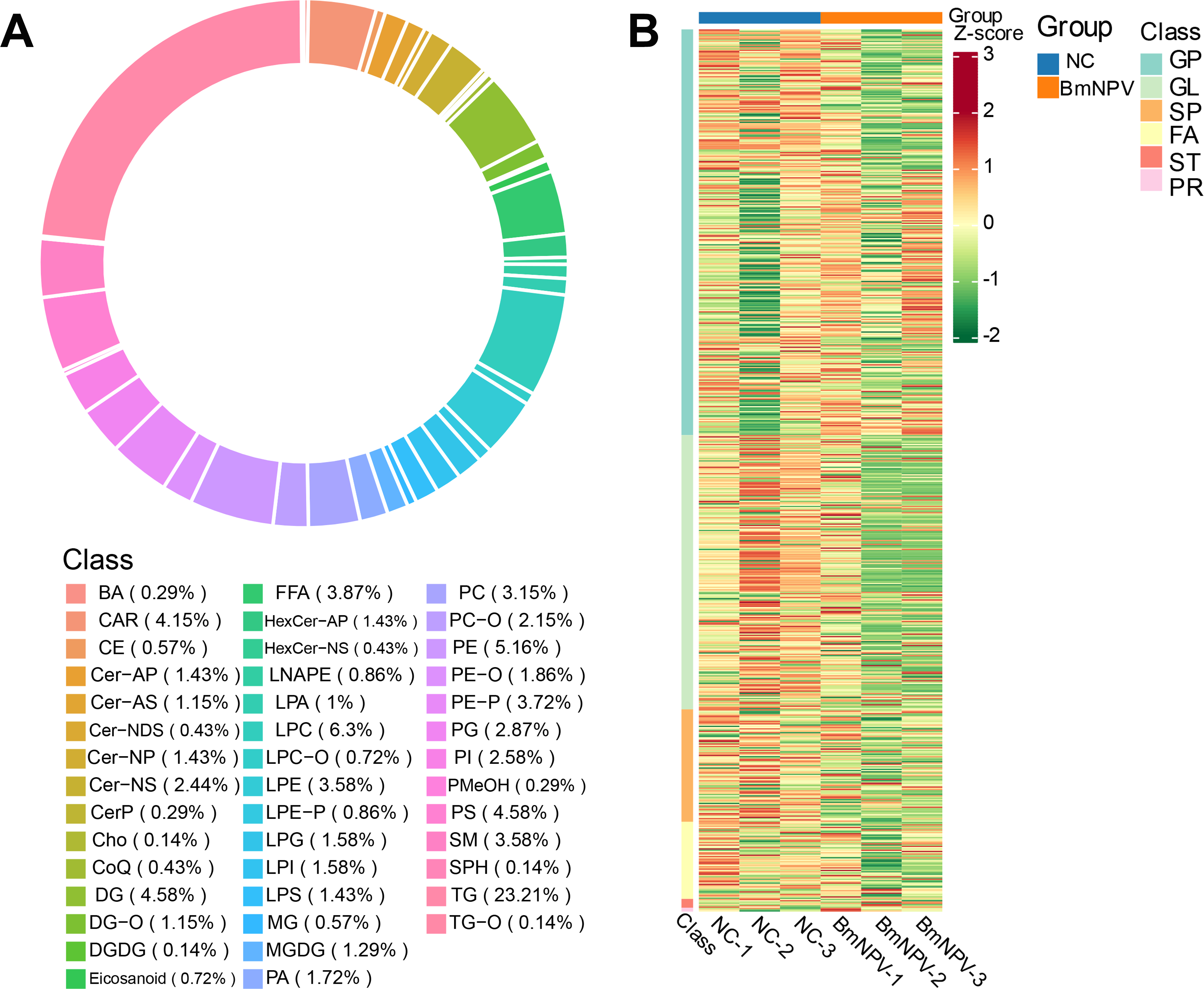

Supplement: S9 Fig — (A) Lipid subclass composition donut diagram. Each color represents a lipid subcategory and the area indicates the proportion of that category. For classification of lipids see [91] and [92]. (B) Cluster analysis of detected lipids. After unit variant scaling, heat map analysis was carried out on all samples. Differential values after normalization (Z-scores) are presented as color intensity (red represents increased abundance, green represents decreased abundance). The different classes of lipids are indicated (GP, glycerophospholipids, GL, glycerolipids; FA, fatty acids; SP, sphingolipids; ST, sterols). (TIF) [file ppat.1013701.s009.tif]

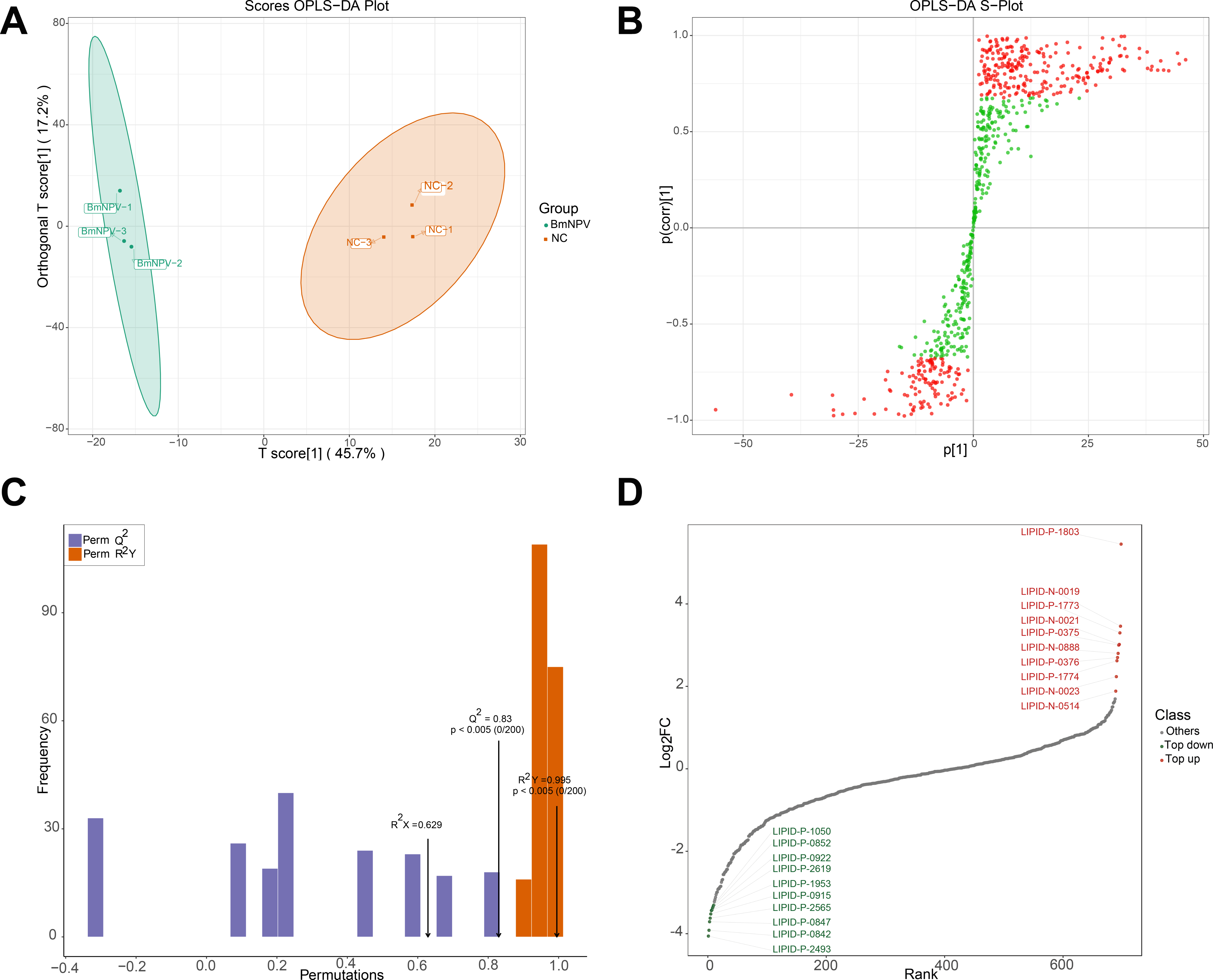

Supplement: S10 Fig — (A) OPLS-DA score plot. The abscissa and ordinate represent orthogonal principal components. Samples of different groups (NC, negative control; BmNPV, infected) are shown indifferent colors. (B) OPLS-DA S-plot. The abscissa and ordinate represent the covariance and the correlation coefficient, respectively, between the principal components and lipids. The closer the lipids are to the upper right corner and lower left corner, the greater the difference. Significantly, red points indicate that the Variable Importance in Projection (VIP) value of these lipids is greater than 1 and green points indicate that the VIP value of these lipids is less than or equal to 1. (C) OPLS-DA verification diagram. The values of R2X, R2Y (explanation rate of the X and Y matrices, respectively) and Q2 (representing the predictive ability of the model) are plotted. The abscissa represents the model R2X, R2Y and Q2 values and the ordinate is the frequency of the model classification effect. Permutation tests are performed to determine the predictive capability of the OPLS-DA model. Excellent values (close to 1) are obtained for R2Y and Q2 together with highly significant P value. (D) Dynamic distribution chart of lipid content differences. The cumulative number of substances arranged in ascending order is plotted against the logarithmic value of the fold difference. The green and red points represent the top 10 substances that were downregulated and up-regulated, respectively. (TIF) [file ppat.1013701.s010.tif]

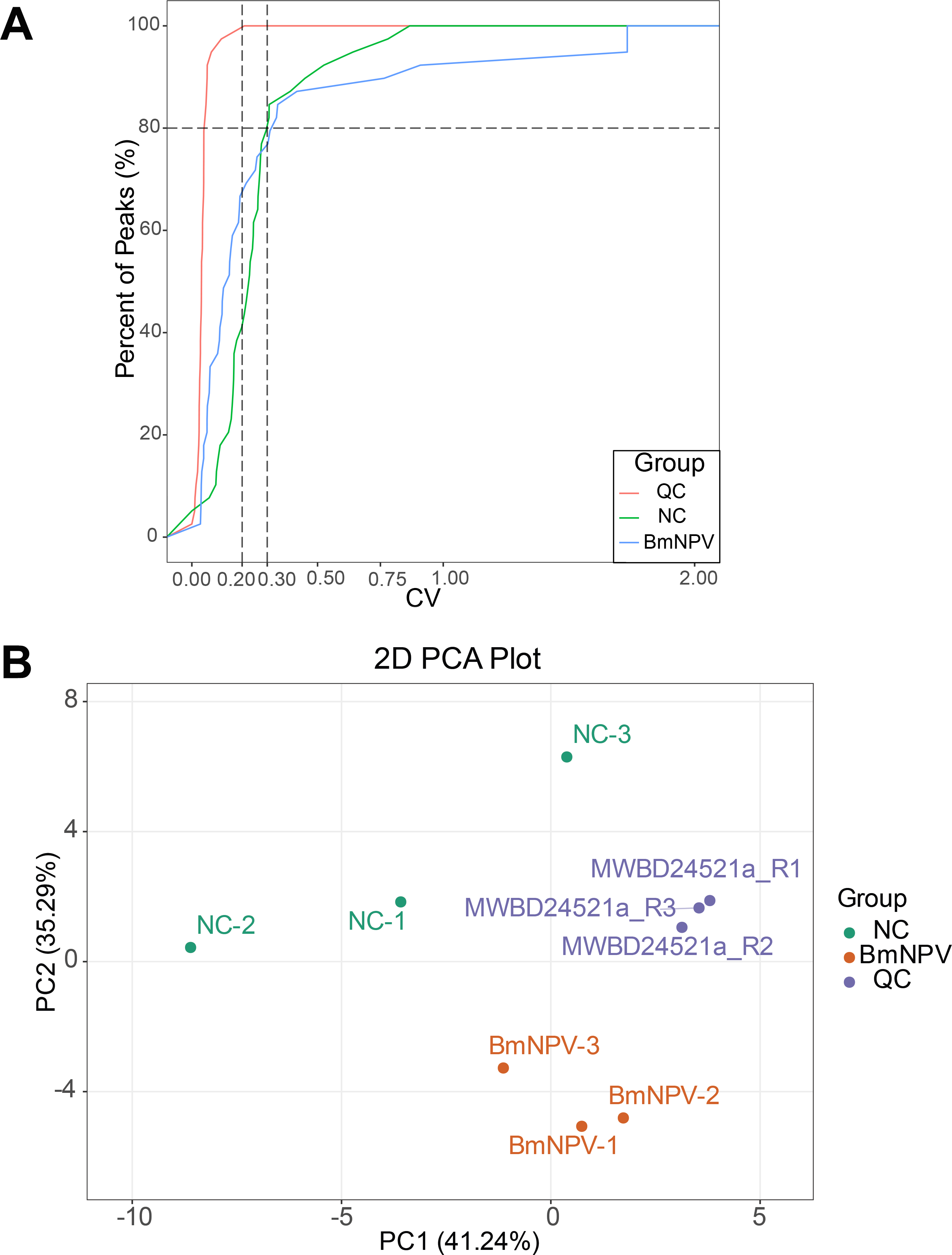

Supplement: S11 Fig — (A) Coefficient of variation (CV) distribution chart of each group of samples. The abscissa represents the CV value and the ordinate represents the proportion of the total number of substances. Different colors represent different grouped samples (NC, negative control; BmNPV, infected; QC, quality control). (B) PCA score plot of mass spectrometry data of each group of samples. PC1 and PC2 represent the first and second principal component, respectively. The percentage represents the explanation rate of the data set by the principal component. Different colors represent different grouped samples (NC, negative control; BmNPV, infected; QC, quality control). (TIF) [file ppat.1013701.s011.tif]
