# Supplementary material for: Chemosensory protein 3 is a brain host factor for the induction of enhanced-locomotory activity in the BmNPV-silkworm infection model
Source: PLoS Pathog. 2025 Dec 1;21(12):e1013701. doi: 10.1371/journal.ppat.1013701 (PMC12688159; doi:10.1371/journal.ppat.1013701)
Supplement: S4 File — (DOCX) [file ppat.1013701.s015.docx]

Supplementary file 4**.** Best structural templates of BmCSP3 based on their sequence identity and coverage

| **Chemosensory Protein** | **UniProt ID** | **Sequence Identity %** | **Coverage** | **Template**  **PDB ID** | **Method** | **Resolution** |
| --- | --- | --- | --- | --- | --- | --- |
| *Bombyx mori* CSP1 | Q8MMK7 | 52.4 | 0.94 | 2jnt | NMR^a^ | *n.a.* |
| *Mamestra brassicae*  CSPA6 | Q9NG96 | 54.3 | 0.96 | 1kx8 / 1kx9 | X-ray^b^ | 2.80 Å / 1.65 Å |
|  |  |  |  | 1n8u / 1n8v | X-ray^c^ | 1.80 Å / 1.39 Å |
|  |  |  |  | 1k19 | NMR^d^ | *n.a.* |
| *Schistocerca gregaria*  CSPsg4 | O76476 | 52.4 | 0.96 | 2gvs | NMR^e^ | *n.a.* |
| *Spodoptera litura*  CSP8 | A0A0P0EUM0 | 53.4 | 0.94 | 7e8l | X-ray^f^ | 2.30 Å |
